# The No-Cloning Life: Uniqueness and Complementarity in Quantum and Quantum-like Theories

**DOI:** 10.3390/e25050706

**Published:** 2023-04-24

**Authors:** Arkady Plotnitsky

**Affiliations:** 1 Literature, Theory, Cultural Studies Program, Purdue University, West Lafayette, IN 47907, USA; plotnits@purdue.edu; 2 Philosophy and Literature Program, Purdue University, West Lafayette, IN 47907, USA

**Keywords:** complementarity, consciousness, observation, measurement, no-cloning, uniqueness, the unconscious, reality without realism

## Abstract

This article considers a rarely discussed aspect, the no-cloning principle or postulate, recast as the uniqueness postulate, of the mathematical modeling known as quantum-like, Q-L, modeling (vs. classical-like, C-L, modeling, based in the mathematics adopted from classical physics) and the corresponding Q-L theories beyond physics. The principle is a transfer of the no-cloning principle (arising from the no-cloning theorem) in quantum mechanics (QM) to Q-L theories. My interest in this principle, to be related to several other key features of QM and Q-L theories, such as the irreducible role of observation, complementarity, and probabilistic causality, is connected to a more general question: What are the ontological and epistemological reasons for using Q-L models vs. C-L ones? I shall argue that adopting the uniqueness postulate is justified in Q-L theories and adds an important new motivation for doing so and a new venue for considering this question. In order to properly ground this argument, the article also offers a discussion along similar lines of QM, providing a new angle on Bohr’s concept of complementarity via the uniqueness postulate.

## 1. Introduction

This article considers a rarely discussed aspect, the no-cloning principle, made here into a postulate and recast as the uniqueness postulate, of mathematical modeling known as quantum-like, Q-L, modeling (vs. classical-like, C-L, modeling, based in the mathematics adopted from classical physics) and the corresponding Q-L theories beyond physics. The development of such models and theories has become an extensive and rapidly expanding field of research in psychology, cognitive science, decision sciences, and other human sciences. (The latter term is more common in French but is a convenient shorthand.) I shall refer by a model to the mathematical formalism used by a theory, and by a theory to the overall conceptual architecture (including the model it uses) necessary to account for the phenomena considered. Similarly to that of a theory, the concept of a mathematical model has a long history and diverse definitions. I shall not discuss the subject as such or engage with literature addressing it, which is not necessary for my purposes. The present concept of a mathematical model, while open to further qualifications, is sufficient to accommodate those models that I will consider. Additional comments on the subject are offered in Appendix A. As discussed in Appendix A as well, the history of any theory is accompanied by the history of its interpretations, defined by concepts added to a theory, beginning with those that establish how the theory refers to the phenomena it considers.

The uniqueness principle is a transfer of the no-cloning principle (arising from the no-cloning theorem) in quantum mechanics (QM) to Q-L theories. This principle and the uniqueness postulate to which this principle gives rise will be related to several other key epistemological features of both QM and Q-L theories, such as the irreducible role of observation, complementarity, probabilistic causality, and the arrow of time. This article’s concern with the uniqueness principle and all these features are connected to a more general question, considered by this author previously [1,2]: What are the ontological and epistemological reasons for using Q-L models vs. C-L ones? I shall argue that adopting the uniqueness postulate is justified in Q-L theories and adds an important new motivation and a new venue for considering this question. The uniqueness postulate is assumed in this article as a *postulate* rather than only a *principle*. By a principle I mean something that serves as a guidance for, rather than, as in the case of a postulate, an assumption made by a theory or interpretation. In order to properly ground this argument, the article offers a discussion along similar lines of QM, thus also providing a new angle of Bohr’s concept of complementarity via the uniqueness postulate, in effect assumed by Bohr but not expressly considered by him in the way it is here.

The argument of the article is based in a particular interpretation of quantum phenomena and quantum theory, specifically QM, an interpretation extendable to those of Q-L theories. (This article will be concerned with QM and, marginally, with quantum field theory, QFT, in their standard forms, and will only mention alternative quantum theories, such as Bohmian mechanics, in passing.) Quantum phenomena will be assumed to be defined by the fact that in considering them, the Planck constant, *h*, must be taken into account. This definition may require further qualifications (e.g., [3], pp. 37–38). These qualifications are, however, not germane for this article. All phenomena considered here involve *h*, which is essential to the ultimate constitution of nature, assuming that QM and QFT are correct, because *h* reflects the Planck scale, the ultimate scale of this constitution. There are no comparable numerical constants in the phenomena considered by Q-L theories, which leads to important qualifications, discussed later in this article. The present interpretation follows Bohr’s ultimate interpretation developed by Bohr in the late 1930s and based, in addition to complementarity, on a new concept introduced around 1927, that of (quantum) phenomena. That Bohr revised his views several times requires one to specify to which version of his interpretation one refers. I shall do so as necessary, while focusing here on his ultimate interpretation, unavoidably in the present interpretation of this interpretation, because this interpretation, or Bohr’s other interpretations, can be interpreted differently. Unless qualified, “Bohr’s interpretation” will refer to his ultimate interpretation. The designation “the Copenhagen interpretation” requires even more qualifications: it can only be used as an umbrella term for several, sometimes diverging, interpretations, such as those of W. Heisenberg, P. Dirac, J. von Neumann, or still others. For this reason, this term will be avoided here. The present interpretation contains additional features, in particular the Dirac postulate, not found in any of Bohr’s interpretations. These interpretations also do not consider the no-cloning theorem, discovered two decades after Bohr’s death. All Bohr’s interpretations are, however, consistent with the no-cloning theorem and in effect contains the uniqueness postulate, even though Bohr does not use this designation.

Both interpretations, Bohr’s and the present one, belong to the class of interpretations based on the concept of reality without realism (RWR) and designated here as RWR interpretations. In accordance with this concept, these interpretations place the emergence of quantum phenomena beyond representation and knowledge, in which case I shall speak of *weak* RWR interpretations, or even beyond conception, beyond the reach of thought, in which case I shall speak of *strong* RWR interpretations. This article adopts a strong RWR interpretation, as did Bohr in the ultimate version of his interpretation. Unless qualified, the term RWR interpretation will hereafter refer to either Bohr’s ultimate interpretation or the present interpretation.

In these interpretations, the capacity of the mathematics of QM or QFT to predict the outcomes of quantum experiments is beyond conception as well. We know *how* this mathematics works, but we do not and possibly cannot know or even conceive of *why* it works. These predictions are in general probabilistic, regardless of the quantum objects considered, no matter how elementary. This is strictly in accordance with experimental evidence available thus far, which only allows for such predictions. The Dirac postulate, added in the present interpretation, states that the concept of a quantum object is assumed to be applicable only at the time of observation. By contrast, in Bohr’s and most interpretations, quantum objects are assumed to be something existing independently of observation, even if, as in Bohr, quantum objects are still seen as something beyond conception. Technically, Dirac postulate implies that no quantum object can be found more than once or exactly reproduced (“cloned”) by any two observations. As, however, explained in Appendix A, in QM, the assumption that one can speak of the same quantum object in successive observations is a permissible (statistical) idealization.

The concepts of RWR and RWR interpretations were introduced in this author’s previous works, most comprehensively in [3], which, however, did not use the term “the Dirac postulate”, introduced in [4]. There is some overlap between the formulations of the present article and these works, primarily in outlining key concepts, such as complementarity, causality, reality, and reality without realism. I thought that doing so would be beneficial to the reader, who would not need to consult these earlier works to follow this article’s argument. I also added Appendix A that offers further details of these concepts and RWR interpretations of QM. However, beyond refining and changing earlier formulations, this article develops new concepts and a new argument, by focusing on the uniqueness principle, rarely discussed in the Q-L context. One exception, along quantum informational lines, is [5], although the *theory* of consciousness offered there is only epistemologically, rather than mathematically, Q-L, insofar as it does not contain a Q-L mathematical model, even if in principle allowing for such a model.

That we have C-L and Q-L mathematical models in human sciences is not something one would necessarily assume a priori, for the following set of reasons. As a mathematical-experimental science, all modern physics, classical, relativistic, and quantum (the three types of fundamental physical theories currently available), mathematically idealize natural phenomena by disregarding most of their aspects perceived or *cognized* by human subjects. C-L and Q-L theories and especially models, necessarily use, even if less rigidly, this type of reductive idealization. The difficulties of this idealization in C-L and Q-L theories are more apparent if one considers this idealization in informational terms and compares the information considered in these theories with information theory, classical or quantum. The data considered in physics is a form of information, which can be treated as Shannon information, a collection of bits. Shannon information is based in disregarding the semantic content of information (essentially our thinking and language), either classical, which deals with information processing by means of classical physical systems, or quantum, which deals with information processing by means of quantum systems. The mathematical conceptualization and reduction of information in information theory was decisive. It may be seen as an extension of the Galilean reduction of physics to dealing only with nature vis à vis both in nature and thought in Aristotle’s physics. As M. Heidegger observed, in commenting on R. Descartes and Galileo, “modern science is experimental because of its mathematical project” [6] (p. 93). This is the case not only because of the role of quantitative measurements vs. qualitative observation but also because of using the mathematical formalism to represent the physical reality considered, idealized by the Galilean reduction, and, by using this representation, to predict the outcome of experiments.

Information considered in quantum information theory cannot be created by using classical systems and, thus, obeys different principles of processing, and in this sense, it may be seen as quantum information, but *only in this sense*. This is because this information itself, qua information, is classical, Shannon information, observed by human agents with observational instruments used in quantum physics. There is no other information. This information is obtained, by using quantum observational technology, in the experience of human subjects and is communicated by means of language (supplemented by, but not limited to, mathematical or technical terminology) to other human subjects. This communication must be unambiguous to conform to the requirement of modern physics as a mathematical–experimental science of natural phenomena, which are experienced by human agents, helped by observational technology. Using the latter is auxiliary in classical physics or relativity, but is, as explained below, constitutive in quantum physics, making it impossible to neglect this role in establishing quantum phenomena, thereby made always different from quantum objects.

Along with communicating a physical theory itself used in representing or predicting, exactly or probabilistically, the events considered, we must share their verification for this theory to work as a mathematical–experimental science, as any such science has been defined from Galileo on. Science is a human enterprise and as such inevitably involves extra-scientific elements. However, sharing information is human, too. Science, including physics, capitalizes on this aspect of human experience and on the possibility that this communication may be made unambiguous, not the least by using mathematics, which helps us to communicate our theories unambiguously. Linguistic communications may also be unambiguous, necessarily so in describing experimental arrangements in physics or in presenting scientific concepts. In general, however, concepts of ordinary language leave a greater space for ambiguity as concerns the limits of their applicability, as noted by W. Heisenberg, and this ambiguity may affect the use of scientific concepts, which are not always reducible to mathematics [7] (p. 92).

While human sciences are still sciences, the information they deal with is much more difficult to contain by Shannon information. This circumstance complicates the use of mathematical models, either C-L or Q-L, especially in dealing with cognition or *thinking* (a more general category adopted in this article). This is not only because the information supplied by human subjects is difficult to mathematize in an informational–theoretical way, but also because this mathematization cannot be certain to the degree (for all practical purposes, a full degree) it can be in physics. These difficulties do not mean that mathematical models cannot be used in dealing with thinking. They can be and have been. The question instead is the limits of such models, most of which, as might be expected, are probabilistic. The main reason for the rise of C-L and Q-L models in human sciences was the role of probability there. The history of mathematical modeling in human sciences (or elsewhere outside physics, as in biology and neuroscience), has been and remains dominated by C-L probabilistic or statistical models, borrowed from classical statistical physics or, in the last half a century, chaos and complexity theories. During the last decades, however, Q-L models became more prominent in human sciences, as in other fields outside of physics, such as biology and neuroscience. Beginning with A. Tversky and D. Kahneman’s pioneering work in the 1970s to 1980s (e.g., [8,9]), it has been primarily the presence of probabilistic data akin to those of quantum physics that suggested using Q-L models. The grounding of such models (conceptually, ontologically, and epistemologically) has proven to be a more complex question, rarely adequately considered and often disregarded altogether. Addressing this question, however, is, I would argue, necessary if one wants to establish a Q-L theory grounding a given Q-L model. This article is a contribution to approaching this question by arguing for the importance of certain key conceptual and epistemological principles, analogous to those grounding quantum theory and its interpretations, such as the constitutive role of observations, complementarity, and the uniqueness of quantum phenomena.

A qualification is in order. While our thinking is commonly assumed by Q-L theories (and will be assumed here) to be due to the neurological workings of the brain, it is not necessary to assume (and will not be assumed here) that the aspects of human thinking treated by these theories arise from the quantum physics operative in the brain. Q-L theories may apply even if the physics of the brain is physically classical. The brain will be treated here as, *physically*, a “black box”, relating the informational input and output, encountered from either the outside or the inside of a human subject, thus disregarding the physics of the brain, whatever it may be. The character of this information processing will, moreover, be assumed here to be beyond conception, in parallel with strong RWR interpretations of QM, where the black box is that between quantum phenomena registered in observational instruments. There are hypothetical theories that argue that consciousness or thinking is an effect of the quantum physics of the brain, such as, prominently, those by R. Penrose, beginning with [10], and his followers. These theories will be put aside here, in part because, in spite of some suggestions by Penrose and others, there is very little evidence and no worked out theory of how this is possible. One of the difficulties is that Penrose’s argument goes beyond QM or even QFT, because it requires quantum gravity to approach the quantum processes in the brain responsible for consciousness. There is, however, no quantum gravity theory in physics now and the prospects for it remain distant and uncertain. How the physics of the brain makes thinking or consciousness, as we experience it, remains an unanswered question, sometimes referred to, due to D. Chalmers, as “the hard problem of consciousness” [11]. The appeal to consciousness is arguably due to the fact that our manifested inner experience is that of consciousness, and not of the unconscious, *inferred* by theoretical means from our conscious thinking. On the other hand, the unconscious is important to the argument of this article. Just as reality and existence, thinking and consciousness, or the unconscious, will be assumed here to be primitive concepts not given analytical definitions, and given meaning by specifying, as analytically as possible, their features. The unconscious will be assumed to be a form of thinking only manifested in effects that appear, as *present*, in consciousness. Anything one says about the unconscious is inferred from such effects, for example, those manifested in memory or dreams, just as anything existing in matter is inferred on the basis of its effects, beginning with those of the physical world on our perception and thought.

This type of double move of, first, decoupling of the mind from of the brain and, second, giving the unconscious a central role in thinking, was made by S. Freud in establishing psychoanalysis. Freud started his scientific career as a neuroscientist. In the 1890s, he tried to approach thinking, and specifically memory, by grounding them neurologically, a project he eventually abandoned as not feasible given the state of neuroscience then, in favor of considering the mind as a self-contained entity. Psychoanalysis was grounded in this separation. Psychoanalysis was pursued by Freud as a *scientific* project, as a science of the mind, analytically decoupled from the functioning of the brain, while assumed responsible for “mental life”, which, I argue here, is a “no-cloning life” (e.g., [12,13]). This aim of Freud’s project can be ascertained regardless of how one views his other assumptions (some of which remain controversial) or his success in achieving this aim. It is a more complex question whether the mind or part of it, especially the unconscious, is a *mental* black box in the sense of the impossibility of accounting for how the mind, for example, as a Q-L type system, produces such outputs. This impossibility may also place limits on adopting mathematical information theory, classical or quantum, in Q-L theories. While decoupling the mind from the brain, Freud, rather than assuming the mind to be a black box, aimed at accounting, through understanding the unconscious, for the workings of the mind, thus providing a mental ontology of these workings. Some Q-L approaches also aim to do so, although the mental ontologies they consider are different from that of Freud, especially by virtue of their mathematical nature. Mathematics played no role in Freud’s psychoanalytic theory. His account of human thinking was conceptual and narrative, with that of the Oedipal complex as the most famous and most controversial narrative of psychoanalysis. RWR interpretations of Q-L theories preclude any representation or even conception of the ultimate reality considered, and hence any ontology, mathematical, narrative, or other, just as do RWR interpretations of quantum theories.

Whether a representation of the reality considered, or of all this reality, are possible in quantum theory has been intensely debated, beginning with the Bohr–Einstein confrontation, and it remains debated with an undiminished intensity with, it appears, no end in sight. This is not surprising, because the stakes are high: the future of our understanding of nature and thought alike, to which quantum physics brought an entirely new set of possibilities. In strong RWR interpretations, beginning with that of Bohr, the *ultimate* nature of the reality responsible for quantum phenomena is placed, more radically, not only beyond representation but also beyond conception. There is only a representation of quantum phenomena observed with measuring instruments. This article extends this type of interpretation to Q-L theories, by assuming that the ultimate reality (be it material or mental) responsible for cognitive and psychological phenomena is beyond the reach of thought. Bohr ventured some tentative suggestions along these epistemological lines beyond quantum physics, without, however, developing them (e.g., [14], pp. 167–179). The present or Bohr’s interpretation of QM does not exclude realist alternatives (theories or interpretations) in accounting for quantum phenomena or Q-L phenomena in human sciences, and such alternatives have been advanced. It would be difficult to argue that quantum theory or Q-L theories require RWR interpretations, and it is not my aim to do so. I only claim the logical consistency of these interpretations and their accord with the experimental evidence, *as currently constituted*. This is a crucial qualification, assumed throughout this article. New evidence or a new understanding of the existing evidence can make any theory or interpretation obsolete.

The next sections introduce RWR interpretations of quantum phenomena and QM, focusing on the role of an agent’s decision in quantum experiment, complementarity, and the uniqueness postulate. Further details are found in Appendix A, which is referred to as necessary. Section 4 discusses Q-L theories from the perspective on quantum physics it develops, with a special focus on the uniqueness postulate, defining the no-cloning life of thought and thus our life in general because it is indissociable from the life of thought.

## 2. Observation, Uniqueness, and Complementarity in Quantum Theory

This section considers the irreducible role of observation, the uniqueness of each quantum phenomenon or event, reflected in the uniqueness postulate, and Bohr’s concept of complementarity, as the latter appears in Bohr’s ultimate interpretation, where this concept applies to (quantum) phenomena in Bohr’s sense, in accord with the epistemological situation defined by Bohr’s ultimate interpretation. This situation includes the role of the agent’s decision in staging one or another quantum experiment, in juxtaposition to this role, still present but merely auxiliary, in classical experiments.

I begin by summarizing the key futures of quantum theory and of RWR interpretations, based on the concept of reality without realism (RWR). This concept can ground a set of interpretations of quantum phenomena and QM (to which this article is primarily restricted) or QFT, one which is adopted here. It follows Bohr’s ultimate interpretation, but expressly adopts the uniqueness postulate, only implicit in Bohr, and adds the Dirac postulate, not found in Bohr.

The concept of RWR presupposes more general concepts of reality and existence, assumed here to be primitive concepts and not given analytical definitions. By “reality” I refer to that which is assumed to exist, without making any claims concerning the *character* of this existence, claims that define realism, the concept explained below. The absence of such claims allows one to place this character either (a) beyond representation or knowledge, or (b) beyond conception, beyond the reach of thought. The concept of RWR is defined by this placement in, respectively, its weak (a) and strong version (b). I understand existence as a capacity to have effects on the world. The assumption that something is real, including of the RWR-type, is generally inferred from such effects, beginning with those of the outside world (the existence of which is still an assumption) on our phenomenal perception.

Realist physical theories aim ideally to represent the objects that theory considers and their behavior, and predict it, either ideally, exactly, or probabilistically (as in classical statistical physics or chaos theory), by using this representation. I also refer here, as is common, to realist theories as ontological. Although the terms “realist” and “ontological” sometimes designate more diverging concepts, they are usually close in their meanings and will be used, *as adjectives*, interchangeably here. Another term sometimes used for realist theories is “ontic”, coming, as ontological, from the ancient Greek *on* (being). I adopt “realism”, *as a noun*, as a more general term and by an “ontology,” *as a noun*, refer more specifically to the representation or conception of the reality considered by a given theory. Classical physical theories (such as classical mechanics, classical statistical theory, chaos theory, or classical electromagnetism) and relativity (special and general) are realist theories, based on the assumption that one can observe the phenomena considered without affecting them, and as a result, identify them with the corresponding objects. It is this assumption that grounds these theories as realist. Not all realist theories or interpretations are of this type, including in the case of realist interpretations of QM, which are possible. On the other hand, the *identification* between the observed phenomena and objects considered is not possible in dealing with quantum phenomena regardless of interpretations, and hence, also not possible in realist interpretations of QM or alternative theories of quantum phenomena, such as Bohmian mechanics. It is not possible because of the irreducible role of observational instruments in the constitution of quantum phenomena. As Bohr argued already in the Como lecture of 1927, which presented his first interpretation of QM (even though this interpretation retained, ambivalently, some elements of realism), in classical physics and relativity “our … description of physical phenomena [is] based of the idea that the phenomena concerned may be observed *without disturbing them appreciably*” [15] (v. 1, p. 53; emphasis added]. By contrast, “any observation of atomic phenomena will involve an *interaction [of the object under investigation] with the agency of observation* not to be neglected” [15] (v. 1, p. 54; emphasis added). I shall, nevertheless, refer to this role as the Heisenberg postulate, following [4], because, as explain in Appendix A, it was introduced by Heisenberg in the course of his discovery of QM in 1925 (possibly under Bohr’s influence), and was adopted by Bohr as central to all of his interpretations. In these interpretations, especially in Bohr’s ultimate interpretation, the interaction between the object under investigation and the agency of observation *gives rise* to a quantum phenomenon, rather than *disturbing* this object by an observation [15] (v. 2, p. 64). The irreducible nature of this interaction and thus the interference of technology into physical reality in quantum physics opens the possibility of RWR interpretations.

In RWR interpretations, QM does not represent the physical emergence of quantum phenomena. This emergence is placed beyond representation or, in strong RWR interpretations, even conception, as it is in Bohr’s ultimate interpretation or the one adopted here, both of which are strong RWR interpretations. Nor, in either interpretation, does QM represent quantum phenomena themselves, which are represented by classical physics, a key feature of quantum phenomena, introduced by Bohr. This assumption will hereafter be referred to as the Bohr postulate, completing the quartet of key postulates used here—the Heisenberg, Bohr, and Dirac postulates, and the uniqueness postulate [3,4]. QM only predicts, in general probabilistically, the outcomes of quantum experiments, observed classically. As noted, these predictions are in accordance with what is observed: no other predictions are possible, because the repetition of the identically prepared quantum experiments, as concerns the (classical) state of observational instruments, in general leads to different outcomes.

However, the nature of the probabilities used is different from those of classical physics, even in realist interpretations of QM. Specifically, quantum probabilities are nonadditive: the joint probability of two or more mutually exclusive alternatives in which an event might occur is not equal to the sum of the probabilities for each alternative, as it is in classical probability theory. How does QM calculate these probabilities? Although familiar by now, the mathematics of the theory was a radical change from the mathematics previously used in physics, on the following points in particular—(1) the use of complex numbers, (2) noncommutativity, and (3) Born’s rule. Since the publication of J. von Neumann’s classic *The Mathematical Foundations of Quantum Mechanics* [16], QM and QFT commonly use Hilbert-space formalism, which gave QM a more abstract and more rigorous form. There are other current versions, such as those using C*-algebras or category theory, more or less equivalent mathematically to Hilbert-space formalism, which remains dominant, as it is in Q-L models. I now spell out the key features stated above in this version, restricting myself to QM (QFT involves additional complexities):

(1) While all previous physics *fundamentally* used mathematics, such as spaces and functions, over real numbers, ℝ, and was finite-dimensional, QM uses Hilbert spaces over complex numbers, ℂ, which are abstract vector spaces of both finite and infinite dimensions. A Hilbert space possesses the structure of an inner product that allows lengths and angles to be measured, analogously to an n-dimensional Euclidean space, which is a Hilbert space over real numbers, ℝ. I add emphasis on “fundamentally” because classical physics or relativity may use complex numbers, but merely practically, for calculations. Complex numbers do not figure in the final solutions of the equations used or are related to what is observed, and everything that one could observe is always represented by real (technically, rational) numbers, which is also the case in quantum physics.
(2) The second key feature is the noncommutativity of Hilbert space-vectors and operators, known as “observables”, which are *mathematical* entities over ℂ, as opposed to classical physics and relativity, where all observable quantities are represented by commuting functions of real variables. The complex quantities of the formalism are related to *physically* observable real quantities by means of (3).
(3) Born’s or an analogous rule (such as von Neumann’s projection postulate or Lüder’s postulate) establishes the relationship between the so-called “quantum amplitudes”, which are complex quantities associated with Hilbert-space vectors, and probabilities, which are real numbers, by using square moduli (or, equivalently, the multiplications of these quantities and their complex conjugates), which are real quantities. Technically, these amplitudes are first linked to probability densities.

The probabilities involved are nonadditive: the joint probability of two or more mutually exclusive alternatives in which an event might occur is not equal to the sum of the probabilities for each alternative, as in classical probability theory. Instead, they obey the law of the addition of “amplitudes” for these alternatives, to the sum of which Born’s rule is then applied. I shall, for convenience, only refer to Born’s rule from now on. In the simplest case, when ψ is a wave function for a particle in the (position) Hilbert space, Born’s rule says that the probability density function *p (x, y, z)* for predicting a measurement of the position at time *t*_1_ is equal to ${\left|\psi (x,y,z,t_{1})\right|}^{2}$. Integrating over this density gives the probability or (if one repeats the experiment many times) statistics of finding the particle in a given area. Although Born’s or similar rules are connected naturally to the formalism, they are added to rather than contained in it. We do not know why these rules work. Yet, we do not know either why the whole scheme of QM works.

As noted above, the features just outlined do not exclude realist theories of quantum phenomena or interpretations of QM. Quantum phenomena only exclude deterministic interpretations of QM, which would allow for (ideally) exact predictions concerning the behavior of individual quantum systems. However, because the irreducible role of observation, by technological means, in the constitution of quantum phenomena, RWR interpretations preclude both realism and, as a consequence, classical causality, rather than only determinism. The concepts of classical causality and determinism, as well as quantum causality, are discussed in Appendix A. I shall, however, give basic definitions of these concepts here. Classical causality is defined by the claim that the state, *X*, of a physical system is determined, in accordance with a law, at all future moments of time once its state, *A*, is determined at a given moment of time. Determinism (in the present definition) refers to our capacity to make exact predictions, not guaranteed in the case of classical causality, as in classical statistical physics or chaos theory, which deal with systems too complex to predict their behavior deterministically. Quantum causality is defined, as probabilistic causality, by the fact that our decision concerning which measurement to perform establishes the *actual* reality of an event and a *possible* (but the only possible) future course of reality, and by complementarity exclude the possibility of certain alternative states of reality at the time of this measurement and the corresponding alternative courses of reality following this measurement.

In Bohr’s and the present interpretations, as strong RWR interpretations, quantum phenomena and QM are defined by the combination of four features:

(1) The ultimate reality responsible for quantum phenomena, as a reality beyond conception and, as such, *invisible to thought*, a reality, commonly, including in Bohr, identified with quantum objects, which are, by the Dirac postulate, defined (still as RWR-type entities) only at the time of observation in the present interpretation;
(2) The irreducible role of observational technology in the constitution of quantum phenomena, which, by the Heisenberg postulate, preclude a representation or conception of the ultimate reality responsible for quantum phenomena;
(3) Observed phenomena that are created by the interaction between quantum objects and measuring instruments and as such are always *visible to thought* and even available to our immediate phenomenal perception, with the numerical data observed or the observable parts of measuring instruments, described by classical physics, by the Bohr postulate;
(4) The mathematical formalism of QM (cum Born’s rule), probabilistically or statistically predicting the outcomes of quantum experiments, observed, by using observational instruments, as quantum phenomena, without representing the ultimate reality responsible for these phenomena by (1).

Observational instruments, thus, contain both classical, observable, strata of reality and unobservable, invisible-to-thought, quantum strata of reality, which enable this interaction. As explained in Appendix A, my emphasis on visible and invisible, extended to the idea of *visible* and *invisible to thought* (essentially thinkable and unthinkable), follows Bohr’s persistent appeal to the impossibility of visualization of the ultimate reality responsible for quantum phenomena. Bohr’s view, I argue, reaches the concept of invisible to thought in his ultimate interpretation as a strong RWR interpretation, by definition, given that this interpretation places the ultimate reality responsible for quantum phenomena beyond thought.

Quantum phenomena would not be possible without our interaction with nature by means of experimental technology and our specific (human) ways of observing phenomena and thinking about them. This makes quantum phenomena visible to thought or even to our immediate phenomenal perception and consciousness. In any RWR interpretation, the concept of a quantum object is an idealization created in response to our interactions with nature by means of experimental technology resulting in quantum phenomena. The present interpretation goes further by assuming the Dirac postulate, which makes the concept of quantum object an idealization (still of the RWR type) only applicable at the time of observation. Some of the reasons for a adding this postulate are explained in Appendix A and elaborated in [1,2,3,4]. In Q-L theories, this type of postulate is virtually automatic, because one deals with human subjects, each of which is unique, and is thus correlative to the uniqueness postulate there. This is not quite the case in QM, where the uniqueness postulate applies without the Dirac postulate.

At the same time, importantly, the present interpretation does not assume a uniform or otherwise unified character of the ultimate, RWR-type, reality considered in QM, a character only manifesting itself differently in quantum experiments. This assumption would be in conflict with strong RWR interpretations, which preclude any conception of this reality and, hence, that of its unity or oneness, uniform or not. It is true that, as inconceivable, this reality cannot be assumed to conform to any concept of multiplicity either. In other words, this reality cannot be conceived either as single or as multiple. Accordingly, the situation is as follows. While each time unthinkable, this reality makes each quantum phenomenon, or by the Dirac postulate, each quantum object, as an effect of this reality, individual and unrepeatable, unique. Each quantum phenomenon manifests the inconceivable nature of this reality (or of the corresponding quantum object, which remains beyond conception) each time one encounters this reality through its effects. One can always repeat the setup of a given measurement, because this setup can be classically controlled. Not so, however, as concerns the outcomes of thus repeated measurements. Such outcomes are ideally the same and are predictable ideally exactly in classical or relativistic experiments dealing with individual or simple systems. Probability only enters when the systems considered have a great mechanical complexity to be handled deterministically. By contrast, in identically prepared quantum experiments the outcomes will in general be different no matter how elementary the quantum objects considered. By “identically prepared” I refer to the states of the observed parts of measuring instruments, which states are repeatable because they are described classically. Observable outcomes of identically prepared quantum experiments are not repeatable.

Eventually Bohr adopted the term “phenomenon” to refer strictly to what is observed in measuring instruments, as effects of their interaction with quantum objects. As he said:

I advocated the application of the word phenomenon exclusively to refer to *the observations obtained under specified circumstances*, including an account of the whole experimental arrangement. In such terminology, the observational problem is free of any special intricacy since, in actual experiments, all observations are expressed by unambiguous statements referring, for instance, to the registration of the point at which an electron arrives at a photographic plate. Moreover, speaking in such a way is just suited to emphasize that the appropriate physical interpretation of the symbolic quantum-mechanical formalism amounts only to predictions, of determinate or statistical character, pertaining to individual phenomena appearing under conditions defined by classical physical concepts [describing the observable parts of measuring instruments].

[15] (v. 2, p. 64; emphasis added)

As defined by “*the observations* [already] *obtained under specified circumstances*”, phenomena refer to events that have already occurred and not to possible future events, predicted by QM. This is the case even if these predictions are ideally exact, which they can be in certain circumstances, such as those of the Einstein–Podolsky–Rosen (EPR)-type experiments. The reason that such a prediction cannot define a quantum phenomenon is that a prediction for variable *Q*, as a Hilbert-space operator (for example, that related to a coordinate, *q*), cannot, in general, be assumed to be confirmable by a future measurement, in the way they can be in classical physics or relativity. This is because one can always perform a complementary measurement, that of *p* (the momentum), which is, by Bohr’s complementarity (explained below), mutually exclusive with that of *q.* As such, it will make any value predicted by using *Q* undetermined by the uncertainty relations ∆*q*∆*p* ≅ *h* (where *q* is the coordinate and *p* is the momentum in the corresponding direction), which in principle preclude associating a physical reality corresponding to a coordinate *q* when one measures *p* exactly [3] (pp. 210–212). Hence, one can never speak of both variables unambiguously, even if they are associated with measuring instruments, while any reference, even to a single property of a quantum object considered independently is ambiguous. In classical physics, this difficulty does not arise because one can, in principle, always define both variables simultaneously and unambiguously speak of the reality associated with both variables and assign them to the object itself. By contrast, in any quantum experiment, one deals with a system containing an object and an instrument, which by interfering with this object precludes considering the latter independently. In the present view, moreover, an object is only definable at the time of observation by the Dirac postulate. Thus, in considering a quantum phenomenon, there is always a discrimination between an object and an instrument, and yet an impossibility of physically separating them. This impossibility defines what Bohr called the indivisibility or wholeness of a phenomenon.

While thus, applicable to quantum phenomena, Bohr’s interpretation and the present interpretation, the central concept, defining all modern physics, prior to quantum theory, that of “measurement”, become no longer applicable to the ultimate reality responsible for quantum phenomena. The idea of measurement is a remnant of classical physics and the history that shaped it, beginning with ancient Greek thinking and the rise of geometry, geo-*metry*. In Bohr’s and the present interpretation, by the Heisenberg postulate, a quantum measurement *does not measure* or, in the first place, *is not an observation of* any property of the ultimate reality responsible for quantum phenomena, a property that this reality would be assumed to possess before or even during the act of observation. The concept of observation requires a redefinition as well. An act of observation in quantum physics establishes, *creates,* quantum phenomena by an *interaction* between the instrument and the quantum object. This view also gives a central significance to the category of event, as defining a new, and in turn unique, physical situation each time. Every event of observation radically transforms the situation and redefines the possible future vis à vis the preceding events, no longer meaningful for predictions concerning the future from this point on. In each sequence, moreover, one deals with a *quantum* Markov chain, defined by the fact that the probability of a future event is defined only by the state of things at present and not the preceding history. A quantum Markov chain replaces the standard (additive) probabilities law of classical Markov chains with nonadditive quantum probability laws. As indicated, one can also change one’s decision and perform an alternative measurement instead of the one predicted. QM becomes a theory of transition probabilities between, each time unique, events, defined by experimental technology and our decisions on which experiment to perform. What is observed can then be measured classically, just as one measures what is observed in classical physics. In speaking of “quantum measurement”, I refer to this whole process.

This process must be taken into account in considering the *concept* of complementarity as this concept is defined by Bohr when it applies in quantum physics. As defined more generally, complementarity is characterized by (A) a mutual exclusivity of certain phenomena, entities, or conceptions; and yet (B) the possibility of considering each one of them separately at any given point; and (C) the necessity of considering all of them at different moments in time for a comprehensive account of the totality of phenomena that one must consider in quantum physics.

A paradigmatic example in quantum physics is the mutual exclusivity of *exact* simultaneous position and momentum measurements in view of the uncertainty relations, ∆*q*∆*p* ≅ *h* (where *q* is the coordinate and *p* is the momentum in the corresponding direction). The uncertainty relationships are experimentally confirmed laws independent of any theory, with which QM is, however, fully in accord. Both variables can be simultaneously measured inexactly. However, at any moment in time, one can (this is always possible) perform either one exact measurement or the other, and, in Bohr’s or the present interpretation, define one or the other corresponding phenomenon, but never both together, in accordance with (A). On the other hand, one can always decide and thus has freedom, at least, as explained below, sufficient freedom, of choice to perform either measurement, as reflected in (B) and (C).

It is worth noting that wave-particle complementarity, with which the concept of complementarity is often associated, had not played a significant, if any, role in Bohr’s thinking, especially after the Como lecture of 1927 [15] (v. 1, pp. 52–94). Bohr was always aware of the difficulties of applying the concept of physical waves to quantum objects or assuming that both types of behavior, particle-like and wave-like, pertain to the same individual entities, such as each photon or electron, considered independently. Bohr’s ultimate solution to the dilemma of whether quantum objects are particles or waves was that they were neither, any more than anything else, in accord with RWR interpretations. Either “picture” may legitimately refer to one of the two mutually exclusive sets of discrete individual effects, described classically, of the interactions between quantum objects and measuring instruments. The effects may be particle-like, which may be individual or collective, or wave-like, which are always collective, composed of discrete individual effects. An example of the latter are interference effects, composed of a large number of discrete traces of the collisions between the quantum objects and the screen in the double-slit experiment in the corresponding setup, when both slits are open and there are no means to know through which slit each object has passed. If such a knowledge is possible, even in principle, no interference effects are observed, only randomly scattered effects are. These two types of effects involved are mutually exclusive and require mutually exclusive experimental setups to be observed. While these *two sets of effects* are, thus, complementary (with the statistics for each correspondingly predicted by QM), in both cases the properties observed pertain to two mutually exclusive *sets* of discrete phenomena observed in instruments. These properties do not belong to any continuous phenomena or any continuous reality ultimately responsible for (discrete) quantum phenomena. As an RWR-type reality, however, the reality ultimately responsible for quantum phenomena cannot be assumed to be discrete either. In classical physics, wave-like (radiation) and particle-like *objects* or (as they can be identified) phenomena were treated by two mutually exclusive theories, which is not the same as being complementary in Bohr’s sense. The latter must include (B) and (C) as part of the concept, applicable to the same (quantum) objects or the ultimate reality responsible for quantum phenomena, but leading to different phenomena by (A), depending on which setup one decides to use. As Bohr observed, reiterating his argument concerning quantum probability as different from a classical one (discussed in Appendix A):

Just in this last respect [of the renunciation in each experimental arrangement of the one or the other of two aspects of the description of the physical phenomena] any comparison between quantum mechanics and ordinary statistical mechanics,—however useful it may be for the formal presentation of the theory,—is essentially irrelevant. Indeed we have in each experimental arrangement suited for the study of proper quantum phenomena not merely to do with an ignorance of the value of certain physical quantities, but with the impossibility of defining these quantities in an unambiguous way.

[17] (p. 699)

Bohr’s ultimate, strong RWR, interpretation gave complementarity the corresponding epistemology, in accord with both the Heisenberg and Bohr postulates. Complementarity was now applied to quantum phenomena observed in measuring instruments described by classical physics, and not to quantum objects, which, and hence how phenomena come about, was placed beyond conception. According to Bohr: “Evidence obtained under different experimental conditions cannot be comprehended within a single picture, but must be regarded as complementary in the sense that only the totality of the phenomena [some of which are mutually exclusive] exhaust the *possible* information about the objects” [15] (v. 2, p. 40; emphasis added). In classical mechanics, it is possible to comprehend all the information about each object at each moment in time within a single picture because the interference of measurement can be neglected. This assumption allows one to identify the phenomenon and the object under investigation and to determinately establish the quantities defining this information, such as the position and the momentum of each object, in the same experiment. In quantum physics, this interference cannot be neglected. This circumstance leads to different experimental conditions for each measurement on a quantum object and their complementarity, in correspondence with the uncertainty relations, which preclude the simultaneous exact measurement of both variables, which is always possible, at least in principle, in classical physics. The situation, thus, implies two incompatible pictures of what is observed, as phenomena, in observational instruments. Hence, the *possible* information about a quantum object, the information *to be found* in instruments, could only be exhausted by the mutually incompatible evidence obtainable under different experimental conditions.

On the other hand, once made, either measurement, say, that of the position, will provide the *complete actual* information about the system’s state, as complete as possible, at this moment in time. One could never obtain the complementary information, provided by the momentum measurement, at this moment in time, because to do so one would need simultaneously to perform a complementarity experiment on it, which is impossible. By (B), however, one can always decide to perform either one or the other experiment at any given moment in time. Each measurement establishes the only reality there is, and the alternative decision would establish a different reality. Instead of reflecting an arbitrary selection of one or the other parts of a preexisting physical reality (as we would in classical physics, for example, by deciding only to measure the position of the object and not its momentum), our decisions concerning which experiment to perform establish the *single* reality that defines what *type* of quantity can be observed or predicted and precludes the complementary alternative. Hence, parts (B) and (C) of the above definition are important and disregarding them can lead to a misunderstanding of Bohr’s concept, often misleadingly identified with only (A).

Bohr’s complementarity is not only about a mutual exclusivity of certain entities, essential as this aspect of the concept may be, but also about performing quantum experiments and making predictions by human agents, in some of which a mutual exclusivity becomes necessary. That we have a free or, as explained presently, at least a sufficiently free, choice as concerns what kind of experiment to perform is, as noted by Bohr, in accordance with the very idea of experiment in science, including in classical physics [17] (p. 699). In classical physics or relativity, however, this freedom does not matter fundamentally because it only defines which part of the already established reality one decides to consider. At least in principle, all variables necessary for defining the future course of reality, in accord with classical causality, can always be determined at any moment in time, because there is no complementarity or uncertainty relations. By contrast, quantum physics gives this freedom an essential role, reflected in complementarity. By implementing our decision concerning what we want to do, which measurement we perform, we define the character of physical reality and its future course. This determination allows us to make only certain types of predictions and irrevocably exclude certain other, *complementary*, types of predictions. Moreover, each new measurement, M_2_ at a later moment in time *t*_2_, creates, in E. Schrödinger’s apt phrase, a new “expectation-catalog”, enabled by QM (cum Born’s rule) and the data obtained in this measurement for possible future measurements [18] (p. 154). Once performed, this new measurement, as a new unique event, even if one measures the same variable, makes the previous expectation-catalog, defined by a previous measurement, M_1,_ at time *t*_1_ (which could have been used for predicting outcomes of M_2_) meaningless as concerns any prediction after M_2_ is made. In each such sequence, one, again, deals with a *quantum* Markov chain, defined by the fact that the probability of a future event is defined only by the state of things at present and not the preceding history.

Thus, our decision only establishes a future course of reality within a certain range, subject to probabilistic estimates. The reason is that, while, as discussed earlier, one can control the set-up of the experiment, one cannot control the outcome, which also gives the measurement another element of objectivity. Nevertheless, one always has freedom, or, again, at least a sufficient degree of freedom, to make this choice or to change one’s choice and thus a future course of reality, as opposed to, as in classical physics or relativity, follow what is bound to happen in any event, regardless of our decision of what to do. There such a decision can only affect what part of a preexisting reality we would decide to know. By contrast, in quantum physics, it is not a matter of partial knowledge of what is already there, but of creating, by interacting with nature by means of technology, a new (complete) reality, shaping a future course of events. Complementarity captures this situation.

I qualify throughout by “a sufficient degree of freedom” because, it is not always possible to know what shapes our decision or changes it at a given moment in time. So-called “superdeterminism” denies that we ever have any such freedom (which is only apparent), by assuming that the outcome of all events in the universe is predetermined in advance from the Big Bang onwards (e.g., [19]). This view will be put aside because (apart from its other problems) it is realist and classically causal, and hence incompatible with RWR interpretations. On the other hand, there are factors, interior and exterior to our subjectivity, that may limit our freedom of choice, which make the category of decision preferable to that of (free) choice. The reality and its future course are defined not only by the independent physical reality considered (which does contribute to the initial conditions of the experiment) but by local circumstances—scientific, social, psychological, or other—of a given situation. Hence, I argue that it is preferable to speak of a local decision, rather than a choice, especially a free choice. The role of human decision and at least some freedom of choice has figured significantly in the debates concerning quantum foundations, from those concerning the Einstein–Podolsky–Rosen (EPR) experiment, central to the Bohr–Einstein debate, onwards. All these findings would remain in place if the category of free choice is replaced by that of “local decision”. Such decisions do entail a degree of free choice, but their local nature is more crucial.

Consider, as an example, the Alice–Bob scenario, common in quantum information theory and in considering Bell, Kochen–Specker, and Conway–Kochen (“free will”) theorems. These finding also concerns quantum entanglement and correlations, on which I shall comment on in Appendix A. In dealing with an entangled, ERP-type system (*S*_1_, *S*_2_), Alice makes one (*A*) of two possible complementary measurements (*A*, *B*) on *S*_1_, and communicates (by some classical channel) the outcome to Bob. Bob then can measure *A* on *S*_2_, which would exactly confirm Alice’s communication (in effect a prediction). Bob can, however, always, *locally*, decide to perform an alternative measurement, *B*, on *S*_2_, which will render Alice’s information meaningless as concerns *S*_2_. It would, accordingly, be more accurate to speak of Bob’s decision as free relative to Alice’s decision, even if Bob’s decision was compelled by a factor that would not make it free in his location. By the same token, the category of free will is sufficiently justified in the context of observation and measurement, as *relative* to Bob’s decision vis à vis that of Alice. However, in view of both unconscious psychological and social factors involved in any human decision, this category, or at least that of free choice, appears less fitting than that of *local* decision, which may, but does not, guarantee one or another degree of freedom of choice. This situation has, thus, nothing to do with superdeterminism, which would predetermine Alice’s and Bob’s decisions in advance, vs. the always possible independence of their decisions from each other. On the other hand, the concept of locality (no action at a distance) is fully applicable, even if one sees it in terms of a decision rather than a free choice. The concept of local decision is, as will be seen, also important in Q-L theories.

The conceptual structure of quantum measurement, in conjunction with complementarity, leads to the corresponding understanding of the key feature of QM, the noncommutativity of certain quantum variables, such as those associated with the measurements of a momentum, *P*, and a coordinate *Q*: PQ−QP=iℏ (and hence is not zero and PQ≠QP). This formula is also connected to the uncertainty relations, ∆*q*∆*p* ≅ *h*, which are part of the experimental confirmation of QM, given that, as noted, the uncertainty relations hips are an experimentally confirmed law of nature, independent of any theory. In Bohr and the present view, as correlative to complementarity, the uncertainty relationships represent not only the impossibility of *exactly measuring* both variables simultaneously but also as the impossibility of simultaneously *defining* both. Commonly, noncommutativity is seen as relating to the fact that, if one measures two physical properties involved in one order and then in the other, the outcome would in general be different, which is not the case in classical physics. However, the present understanding of quantum measurement, as a creation of quantum phenomenon, is unique each time and offers a deeper view of this noncommutativity and of the difference in the outcomes arising from reversing the order of measuring complementary variables, as discussed in detail in [3] (pp. 118–122). This view is as follows. First, in the experiment with the initial preparation of measuring instruments at time t_01_, one makes the position measurement, M_1Q,_ at time t_11_ and then the momentum measurement, M_2P_, at time t_21_. Then, with the same initial preparation of measuring instruments at time t_02_ (which preparation is possible because we can control the instruments classically), one reverses the order of the quantities one measures, by first measuring the momentum, M_1P,_ at time t_12_ and then the position at time t_22_, M_2Q_. The outcomes of these two measurements (which would be the same in classical physics) will be different. As is, however, reflected in my double indexing, each set of measurements happens at a different set of (equal) time intervals and requires a different quantum object, thus representing two unique sequences of measurement with unique outcomes, in accordance with the uniqueness postulate, which I shall now discuss in more detail, in conjunction with the “no-cloning” postulate, which is a much more recent conception.

The uniqueness is, arguably, a better term, also in the case of the no-cloning theorem as well. The term “no-cloning” was introduced at the time when biological cloning was much talked about, perhaps influencing the designation. The no-cloning theorem is a catchier phrase, as is no-cloning life, especially as a title, which I used for this article. The uniqueness postulate states that every quantum phenomenon, and thus every quantum experiment is unique: strictly individual and unrepeatable. Apart from Bohr’s argument, the situation captured by the uniqueness postulate has rarely been discussed in the foundational literature on quantum physics. One exception, which merits attention and deserves credit is an argument offered in two articles by Aage Bohr (the son of Niels Bohr) and coauthors [20,21]. While, however, their argument provides a rigorous and radical view of quantum uniqueness, their interpretation is not an RWR interpretation. These articles target the use of the idea of particles in quantum theory, including, intriguingly, by (Niels) Bohr, in the latter case mistakenly in my view. The authors disregard Bohr’s understanding, from in the Como lecture, of quantum objects, including elementary particles, as “abstractions”, with “their properties being definable and observable only through their interactions with other systems [measuring instruments]” [15] (v. 1, p. 57). By contrast, Bohr’s concept of “atomicity” (bypassed by these articles), essentially equivalent to his concept of [quantum] phenomenon, reflects the fact that phenomena have features—specifically discreteness, individuality, and indivisibility—generally associated with atomic properties. These properties would, however, no longer apply to quantum objects, or the reality thus idealized, as an RWR-type reality, to which no properties of any kind are assigned. Phenomena are not atomic or particle-like in the physical sense because they consist of a very large number of atoms and hence of elementary particles. It is true that, in contrast to the present view, Bohr adopted the concept of a quantum object, including an “elementary particle”, as an idealization applicable independently of measurement. Even so, he would not ascribe to any quantum object any physical properties, including those associated with any concept of particle, any more than that of wave. As explained above, any “particle” features would, in Bohr’s interpretation (in any of its versions), only apply to certain effects of the interaction between measuring instruments and quantum objects.

The uniqueness postulate refers to both recordings defining a given experiment, with the first providing the initial data and the second used to verify predictions based on these data. Both will be different either if we repeat the whole procedure in the same set of experimental arrangements, or if we build a copy of the apparatus and set it up in the same manner. This is, as explained, always possible because both copies of the apparatus could be controlled classically. By contrast, their interaction with quantum objects cannot be controlled. Bohr refers to “the finite and uncontrollable interaction between the object and the measuring instruments in the field of quantum theory” [17] (p. 700). The statistics of multiple experiments performed (repeatedly) in the same experimental settings will be the same. On the other hand, an individual quantum experiment, either the initial state of the observed part of the apparatus or in the outcome of the experiment, cannot be reproduced with the same outcome, as it is always in principle possible in classical physics, because the interference of measurement can be neglected. All data obtained in quantum experiments are classical, too, by the Bohr postulate and can hence be unambiguously communicated as well. However, none of such data obtained in a given single experiment can, in general, be recreated by a different experiment with the same preparation. This preparation is again, always possible, because the observable state of measuring instruments is classical.

The impossibility of recreating these data and thus the quantum uniqueness postulate are closely related to the no-cloning postulate, due to the no-cloning theorem in the formalism of QM. The theorem forbids the creation of identical copies of an arbitrary unknown quantum state: that is, a quantum system prepared in a state |ϕ〉 unknown to an observer and the information it potentially defines (as classical information obtainable for means of this system) cannot be copied. There is no unitary universal cloning machine that would clone arbitrary unknown quantum states, in contrast to the universal Turing machine, which is classical. (As noted, several authors are credited with the proof [22,23,24]). On the other hand, quantum information can be swapped from one system to another, a feature on which I shall comment in the next section in connection with the uniqueness postulate of human thinking [25] (p. 1895).

As a technical finding based in the formalism of QM, the no-cloning theorem is open to interpretations as concerns its meaning, and its derivations are based on epistemological assumptions, overt or implicit. The no-cloning postulate is given here a strong RWR-type interpretation. Less radical epistemological assumptions, however, for example, a weak RWR-type interpretation, or even some forms of realism, may also allow one to formulate a no-cloning postulate, grounded in the no-cloning theorem. Such differences may be subtle, but they may also be more important the subtler they are.

Consider, as an example, J. Bub’s form of the no-cloning view [26], based in I. Pitowski’s Bayesian analysis of quantum probability [27]. Bub refers to his view as “a *Bohrian* position, … reformulated information-theoretically” [26] (p. 241, emphasis added). A Bohrian position may, however, not be the same as that of Bohr and is not in this case. Bub juxtaposes this view to Einstein’s view of QM as an incomplete theory, insofar as it does not represent, in a realist and classically causal way, the behavior of individual quantum systems (or vs. Bohm’s view, defined by Bohmian mechanics). According to Bub, in a complete theory (thus defined) “no cloning principle is not fundamental and unrestricted cloning is possible in principle, and what prevents cloning in certain cases is some feature of the dynamics, so that there is a dynamical explanation for the fact that cloning in these cases is, as a matter of fact, impossible or practically impossible” [26] (p. 241). As I explain below, while Bub’s position is closer to that of Bohr than that of Einstein, it is not *Bohr’s position* “merely reformulated information-theoretically”, at least, in the present interpretation of Bohr’s interpretation as a strong RWR interpretation. According to Bub:

In a ‘no cloning’ world, …, no complete dynamical account of a measurement process is possible in general: ultimately, a measuring instrument in a quantum measurement process simply acts as a source of classical information, i.e., it produces a probability distribution over distinguishable measurement outcomes, and how the individual outcomes come about is not subject to further dynamical analysis. … To sum up: if ‘no cloning’ is accepted as a fundamental principle, then our world must be such that there is no dynamical account of the individual occurrence of the outcome of a quantum measurement, which is to say that the world is ‘irreducibly statistical.’ But the impossibility of a dynamical account here does not entail that there can be no actually occurring measurement outcomes or actually occurring events. Rather, we begin with a space of possible events that the quantum theory represents as structured in a particular (non-Boolean) way.

[26] (p. 242)

This view is close to Bohr’s view or the present view *as concerns no-cloning or quantum uniqueness*, but *not as concerns epistemology*. First, Bub’s view allows for the assignment of *properties* to quantum objects, at least at the time of measurement, but, it appears, independently as well. This is not the case in Bohr’s ultimate, strong RWR, interpretation, in which all physical properties considered are only those (defined classically) of the observable parts of measuring instruments. In the present view, moreover, by the Dirac postulate, quantum objects are idealizations (still of the RWR type, so that no properties can be assigned to them), applicable only at the time of measurement. This is not something that is, or has ever been, entertained by Bub. Bub also claims here that “if ‘no cloning’ is accepted as a fundamental principle, then our world must be such that there is no dynamical account of the individual occurrence of the outcome of a quantum measurement, which is to say that the world is ‘irreducibly statistical.’” By contrast, Bohr’s or the present interpretation makes no claims concerning the world and does not assume it to be statistical any more than classically causal. Either interpretation only makes claims concerning our interactions with the world, which is a fundamental difference, unless this is what Bub means as well. It does not appear, however, that he does. These *interactions* are irreducibly probabilistic in dealing with quantum phenomena, which tells nothing about the nature of the ultimate reality responsible for them. In this view, there is no “no-cloning world” apart from us. There is only our no-cloning life, part of which is our interaction with nature entailing the no-cloning or uniqueness postulate. This postulate only concerns these interactions by means of experimental technology and the mathematics of QM, within which the no-cloning theorem is formulated. Nature has no “no-cloning theorem” or “no-cloning principle”, only we do, essential as it is in quantum physics. I note in passing that, in comparing Pitowski’s Bayesian view, and QBism, Bub also misreads QBism as a form of instrumentalism, a misreading (especially, if one adopts Bub’s definition of instrumentalism) also found in his more recent work [28] (p. 232, n. 23). The designation QBism was not in use at the time of Bub’s 2007 article cited here, but his assessment is the same on both occasions. (A useful discussion of the QBist vs. Pitowski’s view is offered in [29]).

As noted from the outset of this article, all actual data and hence all information in physics is classical: it is visible to thought or even to our immediate phenomenal perception and, as such, is unambiguously communicable. All actual information qua information obtainable in quantum physics is classical information as well, which is an informational form of the Bohr postulate. The difference from classical information processing is the architecture of this information. While unambiguously describable and communicable as well, this architecture cannot be created and predicted by classical means, but only by using quantum observational technology, allowing this information to be observed and communicated by human agents. In this sense, in the present view, only in this sense this information is quantum information. By contrast, the *way* this information is obtained is invisible to thought, by the Heisenberg postulate, and nothing about this “way” can be unambiguously communicated. Hence, Bohr speaks of “the essential ambiguity involved in a reference to physical attributes of objects when dealing with phenomena where no sharp distinction can be made between the behavior of the objects themselves and their interaction with the measuring instruments” [15] (v. 2, p. 61). On the other hand, what is observed in these instruments can, by the Bohr postulate, be defined classically and communicated unambiguously.

The *way* in which classical information processing works can always be unambiguously communicated, and any case of such processing, rather than only information itself obtained, is, at least in principle, repeatable. Thus, one can, in principle, exactly repeat the same classical experiment, say, in classical mechanics, by making a copy of the same classical object, with (ideally) the same outcome, in the same setup. By contrast, in quantum physics, we can only repeat the same setup, but, in principle, not the outcome, or object, which in the present view is only defined in each observation, as unique as is each quantum phenomenon. Just as the ultimate reality responsible for quantum phenomena (which reality exist independently of observation) quantum objects are unavailable, *invisible*, to thought, while quantum phenomena are available, *visible*, to thought and even available to our immediate phenomenal perception. This availability does not change the uniqueness of each quantum phenomenon, captured by the uniqueness postulate, reflecting, jointly, each quantum object and the quantum phenomenon containing it within, in Bohr’s terms, the indivisible wholeness of quantum phenomena.

## 3. Observation, Uniqueness, and Complementarity in Quantum-like Theories

This article argues that quantum theory, QM or QFT, and Q-L theories share their Q-L ontological and epistemological architecture, including the key postulates considered in the preceding section, in particular the uniqueness postulate. This architecture is given here an RWR interpretation by assuming that an observation is a creation of phenomena by means of the interactions between one or another agency with the ultimate reality considered (such as certain strata of thought, including the unconscious) rather than observing the preexisting properties of this reality, while this reality itself is placed beyond representation or even conception. There are, however, differences between QM and Q-L theories, requiring the adjustments of these postulates and how (strong) RWR-type interpretations work there. The most significant difference for the present argument is as follows. The ultimate reality responsible for the phenomena considered in QM and in Q-L theories is an equation placed beyond the reach of thought in strong RWR interpretations. The nature or rather (given that the nature of this reality is beyond thought) the *structure* of this reality is different, however. The ultimate reality responsible for quantum phenomena or the reality of measuring instruments, where quantum phenomena are observed, is, as a *physical* reality, shared by us. The ultimate *mental* reality considered in Q-L theories is unique to each of us, as well as inaccessible, except to a limited degree, to anyone else, which places the ultimate nature of thought beyond the reach of thought. This difference, I shall argue, affects all the key postulates considered in this article when they are transferred, to the degree possible, from QM to Q-L theories.

As explained, in quantum physics our decisions concerning which experiments we perform, *in all possible cases*, essentially affect, by interfering with reality by means of observational instruments, what may or, by complementarity, may not happen with one probability or another. These decisions define the course of reality, in contrast to classical physics or relativity, where our experiments, in principle, follow what would happen in any event. Thus, in quantum physics, one *decides*, say, at time *t*_1_, which experiment to perform, either the one concerning the values of the position or that concerning the values of the momentum. This decision then enables one, by using QM (cum Born’s rule), to estimate the probability of finding either the position or the momentum of the object within a certain range at a subsequent moment in time, *t*_2_. At the same time, making either measurement in principle precludes predicting any probability for the complementary variable. If one performs two such measurements in sequence, the outcomes will be different depending on their order, because each creates a different, unique measurement situation. As explained in Section 2, doing so requires two different experimental arrangements and two different quantum objects. This fact, underlain by the irreducible role of instruments and the irreducible individuality, uniqueness, of each situation, is a manifestation of complementarity pertaining to both the initial measurements and the alternative sequences they define.

When considering Q-L phenomena, as in cognitive psychology and decision science, say, in experiments based on asking a given question or set of questions, this situation acquires a new form or structure. This is because the *object under investigation* is a *human subject or subjects*, as a system or systems comprising consciousness and the unconscious, a C–UC system. Such a system is unique to each subject and is ultimately inaccessible to any other *human* subject. Only an infinite (such as divine) being could have such an access. As noted from the outset, in the present view one is dealing strictly with mental rather than physical reality, bracketed even though a physical reality, that of our brains, is responsible for this mental reality. In quantum physics, which concerns independent (in the present interpretation, RWR-type) physical reality, one never deals with any decision on the part of *the objects under investigation*, because these objects are not human subjects. Each human subject, defined by a C–UC system makes then a decision, a subject-decision in response to the question asked. This decision is manifested to the subject’s consciousness, although the unconscious of the subject may affect or even define it, thus bringing in a C–UC system. In this case, one can see consciousness as performing a kind of “measurement” on the unconscious as unknown reality, akin to that responsible for quantum phenomena (e.g., [1,2,30]). There have been arguments that consider quantum objects as differently “responding” to different experimental setups, or even possessing something akin to consciousness or memory (also as responsible for the arrow of time in QM). I am disinclined to ascribe any human attributes or forms of action to inanimate nature. It is more reasonable to see the situation in terms of different *types* of interactions between quantum objects (the latter, moreover, being only defined at the time of observation in the present interpretation, by the Dirac postulate) and observational instruments. I add “types”, because, as explained, by the uniqueness postulate, any actual interaction is different, unique, insofar as each leads to a different outcome. If one instantly repeats the same measurement the outcome will be, or is commonly (there are exceptions) assumed, to be the same. Apart, however, from the idealized nature of this assumption, I here refer to a very different repetition, that of the whole set-up from the beginning, *ab ovo* (e.g., [18], p. 161; [16], pp. 213–218, 335).

To illustrate this difference between quantum physics and QL theories, I would like to consider a claim by Z. Wang and G. Busemeyer in their analysis of complementarity in psychology [31]. This analysis proceeds via the experiment, paradigmatic in QL approaches, of asking two questions, “Do you generally think President Clinton is honest and trustworthy?” (which asked by itself tends to elicit the negative answer) and “Do you generally think Vice President Gore is honest and trustworthy?” (which asked by itself tends to elicit the positive answer). These questions are asked in two sets of trials by reversing their orders, which exhibit two statistically different outcomes. I have discussed the case previously in [1], but I would like to reconsider it here through the lens of the uniqueness postulate, not addressed in [1], which leads to clearer and more nuanced conclusions. According to Wang and Busemeyer: “Once we obtain a measurement on say, Clinton, that decision can create a definite position for Clinton, but then the opinion regarding Gore must be uncertain” [31] (p. 2). This does not appear to be correct, or at least this is not sufficiently qualified. The opinion of the subject regarding Gore is uncertain for an outside agent. An outside agent could, in any event, never be certain what the subject under investigation thinks, even if the subject provides as much information as possible concerning this thinking. In fact, a subject cannot be entirely certain about its thinking either, because of the unconscious, which, as discussed below, contributes to the RWR view of the situation. This is a fundamental feature of human interactions. It is akin to that with the ultimate reality in quantum physics, which can never be known to or even conceived by us, but as explained above, is different by virtue of the unique nature of each human subject’s inner reality vs. the shared reality commonly assumed in physics. This feature affects the Clinton–Gore experiment as well. In particular, the opinion concerning Gore may be certain for the subject under investigation, alongside that concerning Clinton.

This makes the situation different from quantum physics, where the type of uncertainty assumed by Wang and Busemeyer is in place because of the uncertainty relationships and complementarity. In considering a complementary situation in quantum physics, one only deals with the decision of the agent concerning which measurement to perform as the initial measurement, say, of variable *p* at time *t*_0_. This decision also determines what kind of predictions one can make concerning future measurements, which can test our predictions, say, for one at time *t*_1_, keeping in mind that observing and measuring, as one can always decide to do, the complementary variable, *p*, at *t*_1_ will irrevocably preclude verifying the initial prediction. As explained earlier, our decision, including the degree of free choice, on which observation to make is a crucial part of Bohr’s complementarity and is correlative to quantum causality. It is difficult and arguably impossible strictly to instantiate this conceptual structure, given its precise and quantitative nature (defined, moreover, by the role of Planck’s constant, *h*) in dealing with human thinking. It is, accordingly, not surprising that this crucial aspect of complementarity is rarely, if ever, considered in attempts to use complementarity beyond quantum theory, including in Q-L theories. These attempts tend to limit themselves, just as that by Wang and Busemeyer does, to considering the mutual exclusivity part of complementarity. To give another instructive example, there are psychological phenomena, such as so-called bistable perception, dealing with such objects as the Necker cube or the Rubin vase, which lead to spontaneous alternatives between mutually exclusive perceptual states, alternatives sometimes associated with complementarity (e.g., [32]). In such cases, however, we do not and indeed cannot make a conscious decision concerning the way we prefer to see such an object. Thus, this is not complementarity in Bohr’s sense of it as a physical concept, which always applies to human *decisions*, still, as I argue here, requiring suitable adjustments in dealing with human thinking. E. Rubin was a friend of Bohr, and some argue that his ideas influenced Bohr’s thinking about complementarity. That may be. However, this does not change the fact that Bohr’s *concept* as such is a different concept defined by all its aspects, including the decision by an agent on which experiment to perform.

It is, nevertheless, possible to transfer, in addition to the mutually exclusive nature of certain phenomena, other aspects of Bohr’s complementarity, and with them, a form of the uniqueness postulate, probabilistic causality, and the arrow of time, to cognitive psychology and decision science, thus bringing them *closer* to quantum physics. This transfer is fully consistent with the (strong) RWR interpretation assumed here. While, unavoidably, limited, this transfer is important. It can be initiated by adjusting, vis à vis Wang and Busemeyer’s claim above, the view of what happens once we obtain an answer concerning either Clinton or Gore, as the first answer in the sequence considered. Before I explain how, I add a few further qualifications, which reflect the limited nature of this transfer.

In the Clinton–Gore experiment or most Q-L experiments, the statistics of the outcomes are at least in part defined by *classically causal* relationships between the two answers, “measurements”, in a given sequence, for well-established psychological reasons. One can expect these answers and their (rough) statistics in advance because of these classically causal relationships. In the case of quantum phenomena, at least in RWR interpretations, there are no classically causal relationships *in any situation*, and if there are, they are much more difficult to establish and are certainly debated. There is no such debate concerning the classically causal psychological relationships just indicated, even if only *in some situations*, but this is sufficient to establish the difference in question. There is no Planck’s constant, *h*, either, which is responsible for the mathematics of QM *in all its aspects*, and which, as noted from the outset, reflects certain fundamental aspects of nature, all the way down to the Planck scale. There is still the question of entanglement or quantum correlations in Q-L theories, which is a whole other subject, beyond my scope here, although I shall comment on it in Appendix A. For the moment, in quantum physics, once one measures one complementary variable, say, the position, no expectations at all are experimentally possible as concerns a future value of the other at any future point. This is true even if one assumes classical causality connecting quantum phenomena, as in Bohmian mechanics. In other words, when one is asked the second question, concerning the momentum, in any given sequences, the *information* carried over from the preceding question, concerning the position, *does not and, in principle cannot,* influence the response to this new question, or the future predictions from this point on. According to Wang and Busemeyer: “When one is asked a subsequent question, information carried over from the preceding question provides a context for the construction of the second and influences the subsequent response” [31] (p. 2). That may be so in psychology and human decision making, including in the Clinton–Gore experiment, but it is never the case in quantum physics, beginning with the fact that, unlike the human subjects under investigation in the Clinton–Gore or other such experiments, quantum objects do not possess information, which is strictly human. No information carried from an earlier position measurement has any relevance for any subsequent momentum measurement. This fact also renders this earlier information meaningless as concerns future predictions, even though the order of two complementary questions would statistically affect the outcome. To reverse this order, however, requires two different experiments on two different quantum objects. Each quantum measurement is unique, and the noncommutativity of QM reflects this uniqueness, including in the case of two *reversed* complementary measurements, which give us different outcomes, in contrast to classical physics. There, these outcomes are the same, because, neglecting, as one can, the interference of observational instruments, both variables can be determined jointly at any moment in time. Even if one measures only one, the other is definable, thus reflecting the fact that one decides to know one or the other aspect of *the same reality*, which is not complementarity in Bohr’s sense. The future course of reality is classically causally predetermined as well. There is no uncertainty relation or complementarity *in Bohr’s sense*. By contrast, in deciding to establish one or the other complementarity phenomenon in quantum physics one defines a new, *unique*, reality and a new unique future course of reality.

On the other hand, the essential individuality, uniqueness, leading to the uniqueness postulate, found in quantum physics may be seen as akin to the essential individuality, uniqueness, of human thinking and of each of its instances, which thus conforms to a form of the uniqueness postulate, the form of “no-cloning life” [1,2,5]. This uniqueness is, I argue, a key aspect of these situations of human thought and decision making, which appears to be underappreciated by Wang and Busemeyer’s discussion of complementarity in either quantum physics or psychology. It is this uniqueness that allows one to bring quantum physics and Q-L theories of human thinking closer to each other, while respecting the differences between them. The parallel is *limited*, but it is meaningful and is correlated with using, arguably necessarily, some form of Q-L formalism (it may not be strictly the same as in QM) in assessing the statistics of responses in the Clinton–Gore or other Q-L experiments.

First, asking the subject under investigation *any question* by an agent may be seen as a quantum-like interference or intervention into the unknown and ultimately unknowable reality of the subject’s thoughts. This intervention elicits a conscious response, observed by the agent, akin to the way observation works in quantum experiment. This interference defines a future thinking of the subject, the subject’s orientation of thought, conforming to the arrow of time, akin to the way an observation would define the future course of reality in quantum physics. Thus, if one asks the question *just about Clinton*, the subject thinking becomes oriented in a particular way, which will shape the subject’s answer, in accordance with this orientation, concerning the subject’s attitude toward Gore, if such a question is asked next. It is not that the subject opinion concerning Gore *necessarily* becomes uncertain, as Wang and Busemeyer contend. This opinion might just not be there at all, or if it is there, either in consciousness or the unconscious, it *might* become reoriented as a result of the question about Clinton. (This is not guaranteed, but the possibility is statistically significant.) The same *type* of structure is in place if the first question is just about Gore, although the specific outcome may be different, because we deal with an alternative situation of observation, mutually exclusive with the first one. These two situations are complementary, in accordance with Bohr’s concept, because one deals with two mutually exclusive possible courses of reality, each of which is complete by itself, rather than merely representing two different parts of the same reality. In other words, while these two questions or answers, concerning trusting Clinton or Gore, are not complementary by themselves, the two alternative sequences in question may be seen as complementary, leading to the statistics obtained. By contrast, in quantum physics, two such sequences are complementary because the measurements of the two variables involved, such as that of the position and that of the momentum, are already complementary in the first place, correlatively to the uncertainty relationships, for which there is no real analogue in Q-L theories, which have no *h*. Noncommutativity alone is not enough for the uncertainty relations or complementarity.

By the same token, just as in quantum physics, the arrow of time, connecting, always unique, events, is strictly respected in the Clinton–Gore experiment, because one only deals with the future course of reality defined by the first question and answer to it. It is, again, crucial that, as is in the case of complementarity in quantum physics, each alternative reflects the only course of reality, unique in turn, there is, rather than a half of the same reality. Of course, in each alternative situation, the agent’s prediction concerning the second question in the order will be different, depending on their order. It also appears that these predictions obey Q-L nonadditive probability and statistics, as do most such Q-L experiments beginning with Tversky and Kahneman’s pioneering work. The observed outcomes will be statistical, because the reversal will, in general, concern different subjects. But then, so they are in quantum physics, where complementarity experiments, either individual or sequential, are performed on different objects as well. In both cases, one deals not with classical causality (or determinism) but with probabilistic causality, correlative to the arrow of time.

The main difference remains the stratification of the situation in psychology or decision making not found in quantum physics. In quantum physics, complementary future gradients of reality are determined by alternative decisions, allowing for a sufficiently free choice to an agent, in interaction, by means of technology (beyond that of our bodies) with physical reality. Any quantum data can only be created by this technological interaction and not otherwise. This decision always defines an alternative, mutually exclusive, reality and the future course of reality, thus combining the mutual exclusivity of the phenomena considered and the role of an agent’s decision. In human Q-L phenomena, an object under investigation, as in the Clinton–Gore experiment, is a human subject, and one deals with a decision by this subject *elicited* by the question coming from an agent. This question, too, can be defined by a decision of those conducting the experiment. This, however, does not change my main point that the observation that defines the corresponding course of reality is made from within the thinking, including possibly the unconscious, of the subject. This decision defines, from within the subject, the ultimate reality considered, similarly to the decision of the human agent performing a quantum experiment, but only similarly rather than strictly in the same way, because in Q-L psychological experiments, this decision may be determined by the question of the agent. The subject does not decide which question to ask but only which answer to give. In quantum physics, quantum objects, by interacting with the instrument in one setup or another, determined by the agent decision, produces an outcome, defining the future course of reality, including in complementary situations, where this future course of reality is mutually exclusive with the complementary alternative, always possible to define. However, quantum objects or nature do not make decisions or answer questions, except speaking metaphorically: only we do. Nature only allows us, as human subjects, to obtain an answer to questions we pose by using very specific measuring instruments, required by quantum experiments. There is no need for such instruments in psychological experiments. Human agents ask (or answer) questions or make decisions, defining, with the help of nature, the state and the future course of reality, and even quantum objects.

The latter qualification is one of the reasons to adopt the Dirac postulate, making the concept of a quantum object applicable only at the time of experiment. It is difficult (even if not entirely impossible) to assume that human subjects, as objects under investigation in Q-L experiments, are only defined at the time when questions are asked (or more generally, only at a given instant or interval of thought), although their responses may be and in the present view are so defined. The uniqueness postulate still applies, and it may be extended to the thinking of each human subject, although human subjects do change their thinking, and this change can be affected from the outside. It is still generally assumed, however, in psychological experiments that human subjects, as thinking beings, exist independently, each defining the reality of their thought. This is the case even if the ultimate nature of each such reality may, as is assumed here, be RWR, just as the ultimate reality (a single reality, even if different each time we encounter it) is responsible for quantum phenomena. This assumption implies that our thought may not be able to represent itself, akin to the way Gödel’s theorems tell us that mathematics (if it is rich enough to include standard arithmetic) cannot represent itself mathematically.

Is, then, this ultimate (RWR) nature of the reality of thought the unconscious? It is possible and tempting to *think* so. I would like, however, to take a more stratified view of this reality, following [1,2]. Considering the unconscious is uncommon in mathematical Q-L modeling. One exception, without, it appears, assuming the RWR view, is Khrennikov’s argument [30], in which consciousness is viewed in terms of measurements performed on the unconscious, as decisions made by … well, by what? This is already a difficulty, because one might argue, as Freud and others (e.g., Marcel Proust) did, that at least some of such decisions are in fact made by the unconscious. Either way, a human subject, at least part of it, thus also contains a set of interior measuring instruments which produces each such outcome. This is a plausible argument, considered by the present author in detail in [1,2]. I would, argue, however, that human thinking, as Q-L in nature, creates, by performing something akin to quantum measurements or (given the present view) observations, *both* conscious and unconscious representations, which are classical, but the efficacity of which is of the RWR type. If one adopts this view, the ultimate nature of the unconscious becomes stratified by containing both RWR-type reality, beyond representation or thought, and classical-type reality, assumed to allow for a realist representation. The ultimate RWR-type, *beyond thought*, nature of the reality of thought is, however, unconscious.

This view thus extends the present understanding of quantum measurement or, in the first place, observation to the case of consciousness and the unconscious, and their interactions. In this understanding, by the Heisenberg postulate, a quantum observation *does not observe or measure* any preexisting property of the reality responsible for what is observed, but instead *establishes* quantum phenomena by an *interaction* between the instrument and this reality, the effects of which are observed as quantum phenomena. Then, what is observed can be measured classically, by the Bohr postulate. In the case of consciousness, the outcomes of our observations upon the unconscious are manifested to our consciousness in classical representations and the corresponding language, through which these outcomes can then be made manifested to an outside observer. Not so in the case of the unconscious, where, the same type of observational process is still operative, creating classical representations, as manifested in dreams, for example. It is a complex question whether our consciousness experiences dreams as such, rather than only consciously recollecting them when we are awake. Dreams do suggest, however, that our unconscious, too, contains classical representations, scrambled by dreams as they may be, vis-à-vis (most of) our conscious experience. There are no analogues of quantum objects identical to each other within the same type, such as electrons, in human thinking. In parallel with or as a form of the Dirac postulate, however, every instance of thought creates a new, unique, object of experience, responsible for the corresponding classical representation in our mind, conscious or unconscious. It is possible to see consciousness primarily as a mechanism that brings representations created by the unconscious into, and make them *present* to, consciousness. Consciousness certainly has this function, perhaps as its primary function, with the unconscious as the dominant thinking agency. It is also possible, however, as just outlined, to take a more stratified view of the unconscious as, in addition to the RWR ultimate reality in it, involving strictly unconscious classical-like representations, with only some of them transferred to consciousness. Other such representations remain unconscious, possibly without ever becoming conscious. Consciousness would, thus, contain both purely conscious representations, such as those linked to short-term memory, and those transmitted from the unconscious, such as those arising from long-term memory, in both cases producing classical-like effects. However, how these phenomenal effects are possible is beyond the reach of thought, just as is how quantum phenomena are possible.

This understanding is close to that of Freud, except that it is underlain by the RWR view of the ultimate (unconscious) reality responsible for all classical-like representational effects, consciousness. As noted from the outset, Freud’s view was realist and, essentially, classically causal. Both consciousness and the unconscious were seen as representable, again, in conceptual and narrative, rather than mathematical, terms, such as those of unconscious drives or the Oedipal complex. By contrast, in the present view, the ultimate unconscious reality responsible for thought, for *all thought*, is beyond representation or even conceptions, is *beyond thought*. This ultimate RWR-type reality may still be material, while all conscious reality is (phenomenally) classical. This view is, again, possible without assuming that this reality is a product of a quantum physics operative in the brain, although, even without this assumption, in Q-L modeling and theories of thinking, one still deals with open biological systems. This may, in principle, mean that one needs to always use the formalism of open quantum systems, whereas in QM itself considering an isolated system can be made precise (even if it is still an approximation) and suffices in most cases. This observation follows that of Schrödinger for all biological systems, in *What is Life?* [33] and is indebted to [34]. While the subject would require a separate analysis, the argument of this article would apply in this case, assuming that one adopts an RWR interpretation, which is equally allowed by isolated and open quantum systems. I only add that our inner experience, while always *inner*, essentially depends on our interactions (material, mental, or social) with the world and would not be possible otherwise. It is part of an open system. Be that as it may on that score, whether the ultimate (unconscious) reality of our mental life, unique each time, is material or mental may be secondary, as Freud noted [13] (p. 11), without adopting the RWR view of this reality.

## 4. Conclusions: Galileo’s Physics, Shared by Us, and Cervantes’s *Don Quixote*, Unique Each Time

As stated from the outset, by separating human and physical reality, the Galilean reduction made modern physics, from classical mechanics (or what we call “classical mechanics” now) to relativity and quantum theory, the mathematical–experimental science of nature, with mathematics coming first in this conjunction. This primacy of mathematics is essentially due to the capacity to use mathematical formalism to represent, in accordance with classical causality, the physical reality considered and, by using this representation, to predict the outcome of experiments. In the case of individual or simple systems, this can, moreover, be done deterministically. Probability eventually became used, but as indicated earlier and discussed in Appendix A, until quantum theory, this use was underlain by classical causality and hence was merely a practical tool. In quantum theory, this representation and classical causality became difficult to maintain, because determinism was no longer possible on experimental grounds even in considering the simplest individual quantum systems, such as those associated with elementary particles. Following QM, such a representation or even a concept how quantum phenomena come about became, in principle, precluded from RWR interpretations. This made probability irreducible, defined by quantum causality as probabilistic causality. QM found a way to use very abstract mathematics, such that of Hilbert spaces over ℂ, to enable probabilistic predictions in accord with what is experimentally observed, thus still enabling QM and QFT to remain mathematical–experimental sciences, with mathematics playing an even greater role in them.

It is, however, rarely, if ever, considered that the Galilean reduction is twofold, in fact threefold, because the mathematical–experimental nature of modern physics is already predicated on a double reduction. The first is defined by only dealing strictly with physical reality, and the second by dealing with a mathematical idealization of natural processes, which requires disregarding those aspects of nature that cannot be mathematically idealized. There is, however, a third reduction. It is based on disregarding the multiple, and in each case unique, realities of each individual subject, and considering only the single physical reality of nature, which our interaction with which can, in principle, be and is assumed to be shared (The many-worlds interpretation of QM does not affect this point, because such shared material reality is retained within each world involved, and there are no connections between these worlds).

This reduction is much more difficult and perhaps ultimately impossible to pursue, except in *relatively* simple cases in psychology, cognitive sciences, and decision science, or elsewhere in human sciences, in short, where we are dealing with human thinking. This difficulty also brings with it those of the second mathematical reduction as well. This situation relates to the hard problem, as discussed in [1,2]. While, however, still dealing with the single ultimate physical reality, including as responsible for quantum phenomena, QM complicates the Galilean reduction in physics by virtue of complementarity. Correlatively to the uniqueness postulate and quantum causality, complementarity defines the state and the future course of reality in two or more alternative ways by the decision of a human agent and, thus, this agent’s individual inner reality. The nature of this decision is determined by factors that are outside the purview of quantum theory itself. This fact, however, still allows quantum theory to keep its mathematical–experimental character in place. It is true that one can use a technological device as an “agent” initiating or registering outcomes of experiments. Any such device has, however, to be set up, including as concerns which measurements to perform, by a human agent or agents, and it cannot make predictions. By contrast, the capacity to so is one of the characteristic features of human thinking, although it might be found in other animals.

It is difficult to expect a comparable effectiveness of mathematics, sometimes puzzling, as famously to E. Wigner, even in natural sciences [35], in human sciences, where the Galilean reduction may only be partially possible. That, as stated from the outset, need not mean that such theories are impossible. In question instead are the conditions and limits of their effectiveness, including in considering C-L vs. Q-L theories. Given the role of the postulates considered here, complementarity, probabilistic causality, and related epistemologically Q-L features in dealing with human thinking, especially if RWR interpretations apply, one might expect Q-L theories to be more effective than C-L ones. Even in simple cases, such as that of the Clinton–Gore experiment, our thinking appears to be able to produce decisions that C-L theories may not be able to handle, even though the issue does not appear to be entirely settled. As Wang and Busemeyer observe:

Unfortunately, it is true that compared to quantum physics, which provides rigorous and precise predictions about physical phenomena, psychological theories involve many more random variables that are hardly controlled, resulting in lower precision in prediction. To be fair, this is a general challenge that could be raised for any theories in the behavioral and social sciences. However, through rigorous model comparison, empirical studies have shown that quantum models provide an elegant new way to specify general and vague verbal theories in psychology, and better explain and predict many phenomena puzzling to classical models, leading to highly testable models (e.g., [36,37,38]).

[31] (p. 4)

A long list of works, some proceeding more along RWR lines, supporting this view can be added (e.g., [39,40,41]). As I have argued here, however, rather than only a greater effectiveness of Q-L mathematical models, there may be deeper epistemological reasons that justify and may even require the use of Q-L *theories*, underlying this mathematics, just as such reasons do in quantum physics. These reasons are defined by the combination of the postulates here considered and the possibility of RWR interpretations of Q-L theories. On the other hand, the richness and complexity of human thinking, and correlatively, its richer and more complex uniqueness in the case of each human subject may limit the range and effectiveness of Q-L theories. This richness and complexity of is captured by T. S. Eliot’s famous lines:

In a minute there is time

For decisions and revisions which a minute will reverse.

(“The Love Song of J. Alfred Prufrock,” ll.47–48)

There are far too many qualia, the existence of which are the source of “the hard problem”, that one can experience in a minute, during which “there is time for decisions and revisions that a minute will reverse.” This process is strictly unique to each human subject and is too complex to be predicted by any mathematical model, as literature, such as Eliot’s poem (only one of many examples) tells us, as discussed by this author in [1,2]. Eliot’s “Do I dare?” is also a decision question, and the poem repeats the question “Do I dare or do I not dare?” several times. Even a second can reverse such decisions and revisions, while at the same time making each unique and unrepeatable. Eliot, including in this poem, undoubtedly made and reversed his decisions many times in choosing many words, reflecting many qualia. When one deals with the like of Marcel Proust’s *In Search of Lost Time* or James Joyce’s *Finnegans Wake*, the play of decisions and revisions becomes immense. But then, so it is in our no-cloning life, each time unique, all the time, and both Proust and Joyce reflect and, in effect, expressly comment on this parallel, as, again, discussed in detail by this author in [2].

I would, however, like to add two points here, not discussed in [2]. The first is the role of the arrow of time, implied by both Proust’s and Joyce’s titles, an implication sadly missed by this author in [2]. Either novel inevitably moves us to our death, while also speaking of the continuity of life in the *wake* of our existence, at least for a while, and hopefully a long while. For life, too, is governed, evolutionarily and otherwise by the arrow of time, and is likely to end at some point. The second point is that about half-way through writing *Finnegans Wake*, Joyce apparently thought of asking his friend James Stephens to complete the book, with Stephens apparently agreeing, in principle [42] (p. 23). Whatever Joyce’s reasons (such as, reportedly, his frustration with the largely unfavorable reception of already published portions of the novel) for this request, which never materialized, the case is intriguing as a reflection of the uniqueness postulate. Stephens (and many others) could have undoubtedly followed Joyce’s “quantum theory” (the term expressly used by Joyce in the novel) and quantum practice of the novel’s composition [2]. However, it would not have been, could not in principle have been, the *same* composition. The uniqueness postulate precludes this. One can, of course, strictly copy the text of the novel, which is informationally classical, but one cannot copy this text as *Joyce’s* text, which Joyce’s scholarship can genetically study as Joyce’s own within certain limits without even containing or reproducing it, because any such study displaces it as well. This copied text can, in this way, only be, as a text, that of the person who copied it, the situation famously explored in another iconic modernist text, Jorge Louis Borges’s *Pierre Menard, the author of Don Quixote*, in which Cervantes’s “original”, copied word for word, becomes a new novel, Pierre Menard’s novel. Perhaps not coincidentally Galileo Galilei and Miquel de Cervantes were contemporaries, and so were Galileo’s physics and Cervantes’s novel, both at the birth we now call modernity, while Borges and Menard were contemporaries of the rise of twentieth-century physics. An example of Menard’s no-cloning writing is:

It is a revelation to compare Menard’s Don Quixote with Cervantes’. The latter, for example, wrote (part one, chapter nine):

... truth, whose mother is history, rival of time, depository of deeds, witness of the past, exemplar and adviser to the present, and the future’s counselor.

Written in the seventeenth century, written by the “lay genius” Cervantes, this enumeration is a mere rhetorical praise of history. Menard, on the other hand, writes:

... truth, whose mother is history, rival of time, depository of deeds, witness of the past, exemplar and adviser to the present, and the future’s counselor.

History, the mother of truth: the idea is astounding. Menard, a contemporary of William James, does not define history as an inquiry into reality but as its origin. Historical truth, for him, is not what has happened; it is what we judge to have happened.

[43] (p. 43)

Menard’s view of truth is not only that of William James but also that of Bohr (whose ideas are sometimes linked to James’s philosophy, although the evidence of any direct connection is tentative at most), for whom each new experiment defines reality and thus history. Reality does not preexist history, it emerges with it, just as physical reality does, as each time is unique, in quantum experiments, and with that difference we can use the same technology to stage them and the same mathematics to predict them, even if only probabilistically. This is, obviously, not a matter of a correct reading of Cervantes. Instead, it is an illustration (even if not intended by Borges) of the uniqueness postulate in human thinking and decision making. Menard arrived at his decision to strictly copy *the text of Don Quixote*, as against Cervantes’s novel that cannot be copied as *Cervantes’s’* novel, a novel that in turn emerges through a huge manifold of decisions in the processes that can never be copied, which is unique to Cervantes. Every reading of a text, or every (re)construction of the author’s experiences that creates it, make it both unique, again, and uniquely ours in its interplay of qualia, conscious and unconscious, a unique event of experience. The novel, reauthored by us, becomes unique each time, as does any work of literature or art.

In the present view, moreover, this uniqueness is constituted by the RWR-type efficacity responsible for classical-like informational effects, thus comprising unknown Q-L states, akin to the unknown states of QM, say, an unknown state vector |ϕ〉 enabling, if it becomes known, probabilistic predictions (via Born’s rule) concerning possible, classical-like, future informational effects. As indicated, in quantum physics, while this information (defined by this architecture as classical information obtainable by quantum means) cannot be copied it can be “swapped.” According to C. Bennett and co-authors, in their pioneering article on quantum teleportation:

Suppose one observer, whom we shall call “Alice”, has been given a quantum system such as a photon or spin-1/2 particle, prepared in a state |ϕ〉 unknown to her, and she wishes to communicate to another observer, “Bob”, sufficient information about the quantum system for him to make an accurate copy of it. Knowing the state vector |ϕ〉 itself would be sufficient information, but in general there is no way to learn it. Only if Alice knows beforehand that |ϕ〉 belongs to a given orthonormal set can she make a measurement whose result will allow her to make an accurate copy of |ϕ〉. Conversely, if the possibilities for |ϕ〉 include two or more nonorthogonal states, then no measurement will yield sufficient information to prepare a perfectly accurate copy.

A trivial way for Alice to provide Bob with all the information in |ϕ〉 would be to send the particle itself. If she wants to avoid transferring the original particle, she can make it interact unitarily with another system, or “ancilla, “ initially in a known state |$a_{0}\rangle$, in such a way that after the interaction the original particle is left in a standard state |$\phi_{0}\rangle$ and the ancilla is in an unknown state |a〉 containing complete information about |ϕ〉. If Alice now sends Bob the ancilla (perhaps technically easier than sending the original particle), Bob can reverse her actions to prepare a replica of her original state |ϕ〉. This “spin-exchange measurement”… illustrates an essential feature of quantum information: it can be swapped from one system to another, but it cannot be duplicated or “cloned”…. In this regard it is quite unlike classical information, which can be duplicated at will.

[25] (p. 1895)

One could, in principle, similarly “send” a human subject in a given Q-L state to another observer, say, in the Clinton–Gore experiment (with the subject’s answers not known to Alice) to Bob, except that, unlike in quantum physics, it cannot be guaranteed that the subject will remain in the same Q-L state, even in such simple cases. On the other hand, it is literally unimaginable, apart from far-fetched science-fiction scenarios, to have anything like an ancilla of this kind that would allow one to “swap” an unknown Q-L state of a human subject, to replicate this state, which would amount to replicating, *exactly*, this human subject. One might plausibly argue that the only such “ancilla” would be repeating the Universe itself from the Big Bang on, which is impossible in turn, assuming, as is common, that the origin of the Universe is a quantum event, and hence is random and as such unrepeatable. If one assigns a quantum state to the Universe itself (I am not saying that one can), this state cannot, of course, be cloned, only “swapped” by means of an ancilla containing the Universe. Note that any such ancilla itself is not copied, but is sent, as it is by Alice in the spin-exchange measurement. Although this argument, more a parable, illustrating the Q-L uniqueness postulate in the case of human thinking, would automatically apply if the workings of the brain as a physical system are physically quantum, it need not imply that they are. One only needs to argue, as I do here, that our thinking is quantum-like in its uniqueness. This argument would apply even if both the workings of the brain are physically classical and our thinking is C-L (and hence unrestricted cloning is possible), because this argument only assumes that the origin of the Universe is quantum. This would make the probability of copying either the brain or the Universe zero, and in any event, below any probability that would allow one to see such an event as possible. This probability would be virtually zero in practice even if everything is classical, but the quantum origin of the Universe changes the principle behind the situation just sketched.

These “cosmological” considerations do, however, tell us how *unique* each of us is. Life is a small bubbling fluctuation on the fabric of the cosmos. However, its development as life is unique, even if comparable biological formations might be more common in the Universe, which is, however, not certain either, and they have a remote chance to create anything akin to us. Each species and then each of us are much smaller bubbling fluctuations in the bubbling fabric of life, which may make any expectations of anything similar elsewhere, let alone any form of “contact” with any such entities, an anthropomorphic or at least biomorphic, fiction. However, the irreducible contingency of life and death at all scales, ultimately of life itself, which will, in all likelihood, die at some point as well, also makes each of us unique, and gives uniqueness to each moment of our experience. What makes each of us unique is our conscious and unconscious inner experience, our dreams and desires, which define *our* thinking, including our capacity for creative thought in science, philosophy, art, or any human endeavor.

## Appendix A. Quantum Theory and Reality without Realism: Supplementary Considerations

This appendix offers supplementary considerations concerning RWR interpretations of quantum phenomena and QM, specifically that of Bohr, in its ultimate version, and the one adopted here, and of the accompanied concepts and postulates addressed in this article.

I comment first on the concepts of model, theory, and interpretation, and the relationships among them. Although often implicit, an interpretation is unavoidable in any workable physical theory, at least, by virtue of establishing the relationships between it and the phenomena or objects it considers, in modern physics, customarily by means of mathematical models. An interpretation of a given theory is, thus, always an interpretation of how the mathematical model or models used by it relate to the phenomena or objects considered. A theory may, however, involve other interpretive aspects, defined either by concepts already established by this theory or by additional concepts, specific to a given interpretation of this theory. One example of such an additional concept, central for this article, is the irreducible role of observational instruments (described, by the Bohr postulate, by classical physics and not by QM) in the constitution of quantum phenomena in Bohr’s or the present interpretation. It might be more precise to see a different interpretation of the mathematical model defined by a given theory as forming a different theory, because an interpretation may involve concepts not shared by other interpretations. What would be shared is the mathematical model used, at least in terms of the essential equivalence or mutual translatability of its different versions. For simplicity, however, I shall speak of the corresponding interpretation of a theory containing a given mathematical model, specifically of one or another interpretation of QM. In classical physics or relativity, there is more consensus on their realist interpretations, albeit not an entirely unanimous one (e.g., [44]). Both will also be seen as realist here as concerns the auxiliary (reducible), rather than constitutive (irreducible), role of observational technology in them. Accordingly, it is acceptable to speak of classical physics or relativity without referring to their interpretations, and I shall do so here. By contrast, the history of QM has witnessed a seemingly uncontainable proliferation of interpretations. It is not possible to survey them here. Even each rubric on, by now, a long list (e.g., the Copenhagen, the many worlds, consistent-histories, modal, and so forth) contains different versions. The case is only slightly less prohibitive in QFT. In any event, here I am only concerned with RWR interpretations, in fact only with that of Bohr in its ultimate version and the one adopted in this article.

It may be useful, to sharpen the contrast with RWR interpretations, to explain realist theories or interpretations, which are commonly representational in character. Such theories or interpretations aim to represent the reality they consider. In modern physics, this is primarily done by mathematized models, suitably idealizing this reality. It is possible, including in quantum theory, to aim for a strictly mathematical representation of this reality apart from physical concepts, at least as they are ordinarily understood, say, in classical physics or relativity. It is also possible to assume an independent structure, defined by properties and relationships among them, of the reality considered, while admitting that it is not possible either (a) to represent this architecture or (b) even to form a rigorously specified concept of it either at a given moment in history or ever. Under (a), a theory that is merely predictive could be accepted for lack of a realist alternative, usually with the hope that a future theory will do better by being a representational theory. (Einstein adopted this attitude toward QM). What, then, grounds realism most fundamentally is the assumption that the ultimate constitution of reality considered possesses properties and the relationships between them, or, as in (ontic) structural realism, just a structure, the more elemental constituents of which are not defined in terms of properties [45]. Such properties, relationships, or structures may either be (ideally) represented or known, or be unrepresentable or unknown or even unknowable, while still assumed to be conceivable, usually with a hope that they will be eventually known or represented. Most realist theories are representational, however. While this outline does not cover *all* forms of realism, which would be impossible, it would, I would argue, apply to most forms of realism in science or philosophy.

Thus, classical mechanics (used in dealing with individual or small systems), classical statistical mechanics (used in dealing, statistically, with large classical systems), chaos theory (used in dealing with classical systems that exhibit a highly nonlinear behavior), or relativity, special and general, are realist theories. While classical statistical mechanics does not represent the overall behavior of the systems considered because their mechanical complexity prevents such a representation, it assumes that the individual constituents of these systems are represented by classical mechanics. In chaos theory, which, too, deals with systems consisting of large numbers of atoms, one assumes a mathematical representation of the behavior of these systems as physically classical. Relativity posed insurmountable difficulties for our phenomenal intuition, because the relativistic law of addition of velocities (defined by the Lorentz transformation) in special relativity,$\;s=\frac{v+u}{1+{\left(vu/c\right)}^{2}}$, for collinear motion (*c* is the speed of light in a vacuum), runs contrary to any possible intuitive conception of motion. Relativity, however, special and then general, still offers a mathematically idealized representation of the physical reality it considers.

All theories just mentioned are based in the assumption that one can observe the phenomena considered without affecting them, and as a result, identify them with the corresponding objects and their independent behavior. This assumption allows these theories ideally to represent this behavior and to predict it, either exactly or probabilistically (as in classical statistical physics or chaos theory), by using this representation. In other words, it is this assumption that makes these theories realist. Not all realist theories or their interpretations are of this type, including in the case of realist interpretations of QM. This *identification* is no longer possible in dealing with quantum phenomena regardless of interpretations, and hence even in realist interpretations or alternative theories (such as Bohmian mechanics) of quantum phenomena, because of the irreducible role of observational instruments in the constitution of quantum phenomena. Although, as discussed in this article, especially central to Bohr, this understanding originates in Heisenberg’s thinking leading him to the discovery of QM, and hence is defined here as the Heisenberg’s postulate. As he said, shortly before completing his paper containing his discovery: “What I really like in this scheme is that one can really reduce *all interactions* between atoms and the external world... to transition probabilities” (Heisenberg, Letter to Kronig, 5 June 1925; cited [46], v. 2, p. 242). It is the probabilistic nature of QM and the way QM predicts these “transition probabilities” that are commonly emphasized, and they are crucial. However, Heisenberg’s appeal to the “interactions between atoms and the external world” is important as well. In Heisenberg’s “scheme”, QM was only predicting, probabilistically, the effects of these interactions observed in instruments without representing how these effects come about. As explained in the present article, this view replaced measurement in the classical sense of measuring preexisting properties of the objects considered with first establishing, by using observational instruments, quantum phenomena, as different from quantum objects, placed beyond representation or even conception. Quantum phenomena could be treated classically, allowing the data observed to be measured classically, the assumption designated here as the Bohr postulate.

One might consider, as a simple example, how predicting the polarization of a photon appears in RWR interpretations. There are two possible outcomes of measurement (after the initial preparation), as individual events: for example, the horizontal state *x* and the vertical state y. Either is observed classically in measuring instruments. In RWR interpretations, one could not say, as it is said sometimes, that before it is measured, the photon is in a superposition of two physical states. In the present view, moreover, the very concept of a photon, while it cannot be observed as such (only the corresponding effect in measuring instruments can) is only applicable at the time of observation by the Dirac postulate. The wave function allowing one to predict either physical state *x* or *y* is written as $\left|\psi \rangle =\alpha \right|X\rangle +\beta |Y\rangle$ with probability amplitudes of |ψ〉 associated with state vector |X〉 given by α and |Y〉 given by β. In a random experiment, the probability of the photon, when its polarization will be measured, to be horizontally polarized is ${\left|\alpha \right|}^{2}$ and to be vertically polarized is ${\left|\beta \right|}^{2}$ by Born’s rule. (I use capital vs. small letters to differentiate Hilbert-space elements, here operators, like *Q* and *P*, associated with predicting the values of measured quantities, like *q* and *p*, observed with measuring instruments.) Actual predictions will involve *h,* which does not appear in these abstract notations but will once they are properly unfolded to make actual predictions possible. That, however, need not, and in RWR interpretations does not, mean that $\left|\psi \rangle =\alpha \right|X\rangle +\beta |Y\rangle$ represents the photon in a superposition of two *physical* states, *x* and *y*. This is because nothing can be said or even thought of concerning what happens between quantum observations, assuming that even the word “happen” applies. What “happens” between observation is invisible to thought and as such is beyond language as well. Only the mathematical state vectors, designated |X〉 and |Y〉 (in capital letters), in the Hilbert space used, are in a linear (mathematical) superposition, with given amplitudes, and not quantum objects or the outcomes of experiments, outcomes registered by observational instruments. The latter can only register one individual (unrepeatable) event or the other, entailing the uniqueness postulate.

As noted in the article, my emphasis on visible and invisible, extended to the idea of visible and invisible to thought (essentially thinkable and unthinkable) follows Bohr’s persistent appeal to the impossibility of visualization of the ultimate reality responsible for quantum phenomena. The latter, again, are defined as effects of the interaction between this reality and our agencies of observation and as such are always visible to thought or available to our sense perception, in the first place, which makes them phenomena. There are numerous invocations of the impossibility of visualizing how quantum phenomena come about throughout Bohr’s writing (e.g., [15] v. 1, pp. 51, 77, 98, 108, 114–115, 118; v. 2, pp. 51, 59, v. 3, p. 22; [47], p. 88). I have considered this aspect of Bohr’s argumentation in [3,4,14]. I would only like to address here the role of the “invisible to thought”, the term, admittedly, not used by Bohr, rather than merely an impossibility of visualization in Bohr’s interpretation, specifically his ultimate interpretation, *in the present interpretation of his ultimate interpretation*, as a strong RWR interpretation. It is possible to read Bohr’s appeal to the impossibility of visualization merely as the inaccessibility of quantum objects and their behavior in our immediate (spatiotemporal) sense perception or intuition. This intuition, it might be added, is equally unable to grasp the mathematical formalism of QM, which is, however, something that is knowable to us, along with the data (classically observed in quantum phenomena) considered. These data are in fact available by our immediate sensible intuition, just as it is in classical physics, *in contrast to how these data come about*. The latter are not representable either by our immediate sensible intuition or the formalism of QM, or available to any conception we can form and thus to our thought in strong RWR interpretations, which includes Bohr’s ultimate interpretation, as the latter is interpreted here. If one adopts this interpretation of Bohr’s interpretation, then, it is possible to speak of the ultimate reality responsible for quantum phenomena as *invisible to thought* rather than being merely beyond our immediate spatiotemporal *visualization*.

As discussed in the article, in order to support and sharpen his ultimate (strong) RWR interpretation, Bohr introduced a new concept, that of “phenomenon”, designed “exclusively to refer to *the observations obtained under specified circumstances*, including an account of the whole experimental arrangement” [15] (v. 2, p. 64). This concept also reflects his assumption of the necessity of unambiguous, and in this sense objective, communication of the outcomes of experiments, ensured by the classical description of the observable part of measuring instruments, and hence of phenomena. This unambiguous communication is coupled to that of the logical and mathematical structure of quantum theory, a combination that brings QM fully in accord with the standard practice of modern physics. As Bohr said:

It is decisive to recognize that, however far the phenomena transcend the scope of classical physical explanations, the account of all evidence must be expressed in classical terms. The argument is simply that by the word “experiment” we refer to a situation where we can tell others what we have done and we have learned and that, therefore, the account of the experimental arrangement and the results of the observation must be expressed in unambiguous language with suitable application of the terminology of classical physics. [15] (v. 2, p. 39)

Our expectations or probability assignments concerning such outcomes may be different, depending on different information we have pertaining to a given experiment, and in this sense, they are subjective or personal. “Personal” may be a better term insofar as these assignments are shaped by things in the world, such as measuring instruments or other human beings, assumed in this article or by Bohr to exist independently and thus to be external to an agent. Things are rarely, if ever, completely subjective, permitting that such exterior factors are interiorized at the time of an assignment of one or another probability to a future event. In life, too, we can have different expectations concerning future events given the information we possess (which may be different for each of us). QM, as a mathematical–experimental science, gives us a precise probability calculus, which we can share, to predict quantum events, which is, again, all it does in the present view. Life rarely gives us such means. At the same time, however, any quantum experiment that has been performed gives a definitive, informationally communicable, outcome, which can be treated classically. An agent cannot control this outcome but can only predict it probabilistically by means of QM (cum Born’s rule). One might be able to decide (although, as explained in the article, this may not be simply a matter of a free choice) which observation or measurement to perform, for example, one or the other complementary observation. One cannot, however, control the outcome of it as concerns which value one obtains, even if one controls the preparation of the instrument that will register that outcome. In addition, one (either the same or another agent) can always perform an alternative, complementary, observation at the end point of the experiment, which will irrevocably disable the original estimate. In classical mechanics, this problem does not arise because one can always measure and predict, in the case of simple systems, deterministically, all variables necessary for accounting, representationally, for the system considered (When a classical system has a great mechanical complexity, its behavior can only be predicted probabilistically or statistically as well. However, the ultimate constituents of such systems would allow for determinism, which is a crucial difference).

The situation just outlined makes a quantum observation or measurement objective in this double sense—the lack of control of an outcome and the possibility of an unambiguous communication of this outcome. In the present view, however, it is only objective in this double sense, rather than in the sense of *objectively* attributing any properties conceived by human thought to nature itself, apart from its existence and, as part of it, human existence. (Even this attribution is still human.) Making an observation or measurement is, by the uniqueness postulate, a unique act or event of creation with a unique outcome. Such an act that can be performed by a particular agent or several agents and as such has subjective or, again, personal aspects, including those shaping our decision concerning this action. Making such a decision is inherent in the very idea of experiment, but doing so works differently in quantum and classical physics (or relativity), where it, generally, allows one to follow what would happens, regardless of our observation, in any event [17] (p. 699). Once a quantum measurement is performed, however, the outcome becomes fixed as a permanent record, part of the archive of physical data, always classical and visible to thought or even available our immediate phenomenal perception. It may be unknown to others, but that is not the same as being subjective, because it can become known to others. It is also true that, as any record, it must still be experienced by somebody to be meaningful. Still, an *act* of observation or measurement is personal (if sometimes determined collectively), but its *outcome* need not be. It can of course be experienced differently by different agents, and in this regard, it is personal or has personal aspects.

The problematics just outlines have been central to quantum Bayesianism (QBism), which emphasizes and is in fact grounded in the subjective aspects (QBists would say “the subjective nature”) of quantum theory, leading the proponents of the approach to claiming of a subjective nature not only (a) of our probabilistic predictions, but also (b) of the outcomes of quantum measurements (e.g., [48,49]). It follows from the preceding analysis that, while (a) a view assumed here as well, (b) is not. In the present view, following Bohr, these outcomes are objective in the sense of being unambiguously communicable. This assumption is correlative to that of the physically classical description of the observable part of measuring instruments and quantum phenomena. QBism (to which the present author is in general sympathetic) would require a more extended treatment to properly assess its claims, which cannot be done here. I merely reiterate that, independently of how this situation appears in QBism, in the present view, while science is a human enterprise, sharing and communicating our estimates of possible events and experiences is also human and doing so is helpful and even unavoidable in human life. Science capitalizes on this fact and on the possibility that the communication involved may be made unambiguous, helped by the use of mathematical symbols, central to modern physics, from Galileo onwards. These symbols, too, or their organization are visible to thought and hence unambiguously communicable, including those of the mathematical formalism of QM or QFT. Mathematics itself, as a discipline, depends on this fact. In classical physics and relativity, however, how the outcomes of experiments come about is fully available, *visible*, to thought as well, and may be assumed to be independent of observation, for all practical purposes, but, in the present view, only for all practical purposes, defined by human agents and agencies, such as science [3] (pp. viii–xix). This is not the case, even for practical purposes, in quantum physics, in strong RWR interpretations. In quantum physics, the role of human agents and experimental technology cannot in principle be neglected, as reflected in the nature of quantum probability, which is no longer due, as in classical physics, to our *insufficient* knowledge of how the phenomena considered come about. At stake in RWR interpretations is the impossibility in principle of any knowledge or even conception concerning how this happens, which makes probability irreducible. The mathematics of QM is visible to thought as well, and as such is unambiguously communicable. However, how what this mathematics predicts comes about is not known. Modern physics gave us new means of dealing with the world by interacting with it by means of experimental technology and mathematics (as a form of thought). In the present view, however, it gave us no more than such means, even in classical physics or relativity, where, the assumption that the theory actually (ideally) represents nature is workable for all practical purposes [3] (pp. xiv–xviii).

There are several reasons for adding the Dirac postulate in QM and even more so in QFT to a strong RWR interpretation, as considered in [3] (pp. 273–306), [4]. The first reason is the fact that, in Bohr’s interpretation, nothing can be said about quantum objects independently from observation. Even an observation only allows us to assign properties to the instruments used, impacted by quantum objects and not these objects themselves. In fact, in any strong RWR interpretation, nothing can be said or even thought about what happens between observations. Need one, then, or even can one have a concept of a quantum object, as existing independently, between observations? A quantum object would, however, still be assumed to be an entity that, as such, would be beyond observation, knowledge, or conception in strong RWR interpretations, but only a something that leaves traces, registered marks, in an observational instrument by having interacted with it, before, it follows, the time of observation, and irreversibly so.

If, however, a quantum object is only an idealization defined by an observation, rather than of something that exists independently, vis à vis the ultimate reality responsible for quantum phenomena, is assumed to exist independently in RWR interpretations, even if it adopts the Dirac postulate. Could one, then, still speak of the same quantum object, say, the same electron, in two successive observations? The case can be given a strictly RWR interpretation, insofar as all these properties are, physically, those of observational devices, impacted by quantum objects, rather than of these objects themselves, still placed beyond representation or conception at the time of observation. Rigorously speaking, under the Dirac postulate, the answer is no. A prediction based on a given measurement and the new measurement based on this prediction could only concern a new quantum object, and not an object that one measured earlier in making a prediction. The Dirac postulate implies that one deals with two different quantum objects, two different electrons, for example. To consider them as the same electron is, however, a permissible idealization in low-energy QM, or low-energy QFT, regimes. This idealization is still statistical in nature because there is always a chance, however small, that the second measurement will not register anything. By contrast, speaking of the same electron in successive measurements in high-energy (QFT) regimes is meaningless. This is because these measurements can register quantum objects of different *types*, say, in the case of quantum electrodynamics (QED) an electron in the initial measurement and a positron, or a photon, or an electron–positron pair, in the next measurement [3] (pp. 279–292), [50]. QFT, thus, supports adding the Dirac postulate to RWR interpretations and is the main reason for designating it as such, because it originates with Dirac’s famous equation for the relativistic electron, which also proved to be that for a positron. There are, however, reasons for adopting the postulate in low-energy (QM) regimes, including the complexities involved in the double slit and other paradigmatic quantum experiments [50]. As discussed in the present article, in Q-L theories, this type of postulate is virtually automatic, because one deals with human subjects, each of which is unique, and is thus correlative to the uniqueness postulate there, which is not the case in QM, where this postulate applies without the Dirac postulate.

Two central concepts defining classical physics and relativity, (classical) “measurement” and (classical) “causality”, become no longer applicable in QM in RWR interpretations. I have discussed quantum measurements in detail in the main body of this article. Here, I shall focus on “causality”. Classical causality, which defines classical physics and relativity, is no longer possible in considering quantum phenomena and hence in QM in RWR interpretations. As noted, by “classical causality” I refer to the claim that the state, *X*, of a physical system is determined, in accordance with a law, at all future moments of time once its state, *A*, is determined at a given moment of time, and state *A* is determined by the same law by any of the system’s previous states. This assumption implies a concept of reality, which defines this law, thus making this concept of causality ontological. Some, beginning with P. S. Laplace, have used “determinism” to designate classical causality. I define “determinism” as an epistemological category referring to the possibility of predicting the outcomes of classically causal processes ideally exactly. In classical mechanics, when dealing with individual or small systems, both concepts become equivalent. On the other hand, classical statistical mechanics or chaos theory are classically causal but not deterministic in view of the complexity of the systems considered, which limit us to probabilistic predictions concerning their behavior. There are several reasons for using “classical causality” rather than just causality, used more commonly for this type of concept. The main reason is that it is possible to introduce alternative, probabilistic, concepts of causality, that are applicable in QM, including in RWR interpretations, where classical causality does not apply (e.g., [3], pp. 207–218). One such concept is assumed in this article as correlative to complementarity. As an instance of probabilistic causality, quantum causality is defined by the fact that our decision of which measurement to perform establishes the *actual* reality of an event and a *possible* (but only possible and probabilistically predicted by QM) future course of reality, and by complementarity excludes the possibility of certain alternative states of reality in this measurement and the course of reality that would be defined by any such alternative.

One can prepare any given state, say, that of a “spin-up”, as manifested in the corresponding measurement, even though one cannot always do so in a single experimental preparation but only by post-selecting the required preparation from several repeated trials. By contrast, the outcome of a measurement cannot be controlled at all, only allowing one to predict the probability or, if the experiment is repeated, statistics of the outcome. The statistics of the outcomes of multiply repeated experiments performed in both such experimental settings will be the same. On the other hand, an individual quantum experiment cannot be reproduced (as it is always possible to do so in classical physics), because the interference of measurement can be neglected or controlled, at least in principle. All data observed in quantum experiments remain classical and can be communicated unambiguously. Unlike in classical physics, however, this data cannot be recreated by a different system, which combines a quantum object (in the present view, a concept only applicable at the time of observation, by the Dirac postulate) and an apparatus, the observable part of which is described classically. This situation, as discussed in the article, embodies the no-cloning theorem.

The probabilistic or statistical character of quantum predictions must, on experimental grounds, hold in interpretations of QM or alternative theories of quantum phenomena (such as Bohmian mechanics) that are classically causal. QM, in RWR interpretations, is not classically causal because the ultimate nature of reality responsible for quantum phenomena is assumed to be beyond a representation or even conception. Classical causality would imply at least a partial conception of this reality. These circumstances imply a different reason for the recourse to probability in quantum theory in RWR interpretations. According to Bohr:

[The] recourse to probability laws under such circumstances is essentially different in aim from the familiar application of statistical considerations as practical means of accounting for the properties of mechanical systems of great structural complexity. In fact, in quantum physics we are presented not with intricacies of this kind, but with the inability of the classical frame of concepts to comprise the peculiar feature of indivisibility, or “individuality”, characterizing the elementary processes. [15] (v. 2, p. 34)

Bohr’s reference to “the inability of the classical frame of concepts to comprise the peculiar feature of indivisibility, or ‘individuality’, characterizing the elementary processes” is subtle. “The classical frame of concepts”, unable to *comprise* the situation in question, may be seen as referring to the concepts of classical physics, and it does include these concepts. However, by this point (in 1949), Bohr’s ultimate, strong RWR, interpretation was in place. In this interpretation, all concepts in principle available to us are epistemologically classical, visible to thought. Hence, no concept at all can comprise the emergence of any quantum phenomenon, even though each such phenomenon as such, as observed, is described classically, by classical physics. This is not contradictory, because what these concepts, *all human concepts*, cannot comprise is the “invisibility, or ‘individuality’, characterizing the elementary processes”. More accurately (as, technically, the word “process” can no longer apply either), they cannot comprise how each individual phenomenon or event comes about in its individuality, in accordance with the *uniqueness* postulate. The “indivisibility” now refers not to indivisible atoms of nature on Democritean lines, but to the indivisibility of phenomena in Bohr’s sense, defined by the impossibility of considering quantum objects independently from their interactions with these instruments. As noted, by this point Bohr also introduced another concept, that of “atomicity”, as different from the Democritean ultimate atomic constituent of nature [15] (v. 2, p. 34). This concept is essentially equivalent to Bohr’s concept of phenomenon, but highlights the indivisibility, individuality, and discreteness of each phenomenon, which are now epistemological, rather than physical, features. This is because each phenomenon refers to a physically complex entity, consisting of millions of (chemical) atoms. Each phenomenon is individual and unrepeatable, unique, and is discrete relative to any other phenomenon.

This nature of quantum phenomena, correlatively, implies the essential randomness of individual quantum phenomena, which defines the difference in the classical vs. quantum recourse to probability. Collectively they may not be strictly random by virtue of one or another form of quantum correlations (such as EPR-type correlations, at stake in Bell’s or the Kochen–Specker theorem), which are strictly quantum and not found in classical phenomena. This randomness is not found in classical physics, because even when one must use probability, at bottom, one deals with individual processes that are classically causal and in fact deterministic. Hence, in classical physics, randomness does not ultimately exist or is assumed not to exist: only probability does. In principle, one can isolate an individual constituent of the structurally complex mechanical system, say, a molecule of a gas, the constituent that is visible to thought and predictable ideally exactly as concerns its behavior. This is never possible in considering individual quantum systems, no matter how elementary. By the same token, such systems or the ultimate reality considered can never be made accessible to thought, which is the main reason why they cannot be assumed to be classically causal or predicted deterministically. Naturally, these remarks only reflect some basic elements of the nature of probability and probabilistic representation in physics, a vast subject beyond my scope (e.g., [51,52]). My main point at the moment is that quantum physics *contains* an essential randomness not found in classical physics, which is at bottom classically causal and, when it comes to the behavior of its elemental individual constituents, deterministic, thus making the recourse to probability a practical, epistemological matter.

One might further distinguish between indeterminacy, as a more general category, and randomness, as a most radical form of indeterminacy, when a probability cannot be assigned to a possible event, which may also occur unexpectedly. Both indeterminacy and randomness only refer to possible future events and define our expectations concerning them. Once an event has occurred, it is determined. An indeterminate nature of events may either allow for assuming an underlying classically causal architecture (which may be temporal) of the physical reality responsible for this nature, whether this process is accessible to us or not, or disallow for making such an assumption. The first case, as just explained, defines indeterminacy in classical physics, such as classical statistical physics or chaos theory, or more radically in considering the so-called algorithmic complexity, such as Kolmogorov complexity, which may not be computable, but only for practical, epistemological reasons. The second is found in QM or QFT in RWR interpretations. It is impossible to ascertain that an apparently random sequence of events, events that occurred apparently randomly, was in fact random, rather than connected by some rule, such as that defined by classical causality, and there is no mathematical proof that any “random” sequence is in fact random. The sequences of indeterminate events that allow for probabilistic predictions concerning them is a different matter, although there is still no guarantee that such sequences are not ultimately underlain by classically causal connections in the case of quantum phenomena. Experimentally, again, quantum phenomena only preclude determinism, because identically prepared quantum experiments, in general, lead to different outcomes. It follows that the claim of quantum randomness can, in principle, be falsified by establishing a classically causal theory or algorithm that reproduces the indeterminate or random data in question, which become no longer indeterminately random. This would also falsify RWR interpretations, which preclude such connections.

Quantum physics, however, only *contains* this randomness, rather than being entirely random, because it allows for probabilistic or statistical predictions (purely random events do not) and especially correlations. QM predicts these correlations, specifically by using quantum entanglement, inherent in the formalism, but at least in RWR interpretations, it does not explain them, any more than it explains how any single outcome of an observation comes about, as discussed in [3] (pp. 253–270). The emergence of either is invisible to thought. It is, then, the combination of the irreducible individuality of phenomena, correlative to the uniqueness principle, and the irreducible, RWR, inaccessibility of the reality giving rise to these phenomena, that is a manifestation of quantum vs. classical probability, and the necessity of an alternative to classical physics, such as QM, that would be able to predict such probabilities.

Occurring, as they can be, between arbitrarily distant events, yet without any action at a distance [3] (pp. 227–272), [17], quantum correlations are a unique feature of quantum phenomena. Their unique nature is supported, even if not strictly demonstrated, by Bell’s, the Kochen–Specker, Conway–Kochen (free will) theorems, and related experimental and theoretical findings, which, along with quantum correlations themselves, have been the main focus of the debates concerning quantum foundations during the last half a century. These correlations are not found in classical physics and, arguably, anywhere else, including in the phenomena considered in Q-L theories, even though the latter by virtue of using the mathematical formalism of QM, do contain entanglement, which is a feature of this formalism, while correlations are that of quantum phenomena. There have been suggestions that something akin to quantum correlations may be found in the phenomena considered in Q-L theories (e.g., [53]), but these suggestions, while stimulating, are too tentative to be sufficiently persuasive, at least to this author.

Quantum correlations, it’s been said, manifest the ultimate magic and mystery of quantum physics. They are magical because, conjured by nature in our interaction with it by means of experimental technology and the mathematics of quantum theory, and underlying many (and perhaps all) famously strange quantum phenomena, quantum correlations are something that was literally unimaginable before quantum physics came about. It is still unimaginable, inaccessible to thought, how they are possible, as reflected in strong RWR interpretations. This fact also makes them and with them quantum physics, mysterious. This is, however, not because there is some mystical agency in charge of this situation, as in so-called mystical or negative theology, which presupposed such an agency, while denying that any humanly conceivable properties could be assigned to it. This mystery is, in Bohr’s words, free from any “mysticism incompatible with the true spirit of science” [15] (v. 2, p. 63), [47] (p. 83). Quantum physics is mysterious without being mystical, a mystery without mysticism. On the other hand, our scientific thinking in quantum physics has plenty of *human spirit* that drives it. This, too, is part of quantum magic, because it allows us, our mathematics, physics, and technology, to do so much that was not possible before quantum theory. Perhaps, we can extend at least some of this magic beyond physics.
